# A checklist of land snails from the west coast islands of Sabah, Borneo (Mollusca, Gastropoda)

**DOI:** 10.3897/zookeys.673.12422

**Published:** 2017-05-12

**Authors:** Chee-Chean Phung, Fred Tuh Yit Yu, Thor-Seng Liew

**Affiliations:** 1 Institute for Tropical Biology and Conservation, Universiti Malaysia Sabah, Jalan UMS, 88400 Kota Kinabalu, Sabah, Malaysia; 2 Sabah Parks, Blok K, Lot 1 - 3, Tkt 1, Sinsuran, Peti Surat 10626, 88806 Kota Kinabalu Sabah, Malaysia; 3 Small Island Research Centre, Universiti Malaysia Sabah, Jalan UMS, 88400 Kota Kinabalu, Sabah, Malaysia

**Keywords:** Endemic species, island biodiversity, Labuan, Malaysia, marine parks, Sundaland

## Abstract

Sabah, situated in one of the world’s biodiversity hotspots, has the largest number of islands in Malaysia with more than 500 of various sizes and degrees of isolation. However, information on the islands’ biodiversity is limited. This study provides an up-to-date checklist of land snail species found on 24 west coast islands in Sabah. A total of 67 species (nearly 20% of the total number of land snail species in the state) representing 37 genera and 19 families is enumerated based on systematic field surveys of 133 sampling plots, BORNEENSIS database records and species checklists published between 2000 and 2016. The number of species on the islands ranges from four to 29. Labuan Island has the highest number of species (29), followed by Tiga Island (25), Mantanani Besar Island (24) and Gaya Island (23). However, the populations of some land snail species may have declined as several previously recorded species on the islands were not found in a recent systematic field sampling. This checklist is provided as a baseline inventory for future island land snail studies and to better inform biodiversity conservation plans of marine parks and other islands on the Sabah west coast.

## Introduction

The island of Borneo is recognised as one of the mega biodiversity hotspots in the world (Myers et al. 2000). Sabah, a state in Malaysia and formerly known as British North Borneo, is situated on the northwestern part of the island. Documentation of the biodiversity in Sabah began in the 19^th^ century with explorations by European naturalists in this region. One of the first comprehensive checklists of land snails was published by Issel (1874) who documented an estimated 100 species from Borneo. In the subsequent decades, explorations and collections of land snails in Sabah were conducted mainly by British naturalists such as A. Everett and H. Cuming. Their explorations resulted in several published reports and descriptions of a number of new species (Pfeiffer 1852, 1863; Godwin-Austen 1891; Smith 1894). In the 20^th^ century, information on Sabah land snails was mainly contributed by Jaap J. Vermeulen through taxonomic revisions of several land snail genera (Vermeulen 1991, 1993, 1994, 1996, 1999). In the 21^st^ century, knowledge on Sabah land snails expanded greatly with the publication of detailed taxonomic revisions of certain land snail groups (Liew et al. 2009; Vermeulen et al. 2015), inventories of Borneo land snails (Schilthuizen and Vermeulen 2003; Schilthuizen et al. 2011; Uchida 2013) and ecological studies (Schilthuizen and Rutjes 2001; Schilthuizen et al. 2002, 2003a, 2003b, 2005; Liew et al. 2008, 2010). To date, the number of land snail species documented in Sabah is approximately 350.

Many of these studies were conducted in ecosystems on the Sabah mainland, covering mountains (Liew et al. 2010), tropical lowland rainforests (Schilthuizen and Rutjes 2001; Schilthuizen et al. 2002; Liew et al. 2008; Uchida et al. 2013) and limestone outcrops (Schilthuizen et al. 2003a, 2003b, 2005; Schilthuizen and Vermeulen 2000). To date, there have been very few systematic surveys on the land snail diversity in the island ecosystem. In the 19^th^ century, several new species from Labuan Island, Tiga Island and Usukan Island on the west coast of Sabah were described (Pfeiffer 1863; Adams 1865; Issel 1874; Godwin-Austen 1889; Godwin-Austen 1891; Fulton 1896). It was only a century later that land snail biodiversity studies were conducted on islands such as the Mantanani Island group on the west coast, Balambangan Island and Banggi Island on the north coast, and Bohey Dulang Island, Tetagan Island, Bod Gaya Island, Sebangkat Island, Maiga Island, Mantabuan Island, Pulau Sibuan and Selakan Island on the east coast (Liew et al. 2008; Schilthuizen et al. 2011, 2013).

Despite these efforts, knowledge on land snail biodiversity on the islands remains inadequate as previous studies covered less than 3% of the estimated 500 islands in Sabah (JUPEM 2005). Of these number, 45 islands located on the west coast of Sabah should be prioritised for a land snail survey since (1) they are close to major cities in Sabah and therefore subjected to more intensive tourism activities and economic development, (2) 11 of these 45 have been gazetted as marine parks, and basic biodiversity knowledge is thus vital for park management, and (3) there is already a land snail species at risk of extinction -*Plectostoma decrespignyi*- from Labuan Island, and this indicates that island land snails are very vulnerable to human activities (Schilthuizen and Vermeulen 2004b).

**Figure 1.**
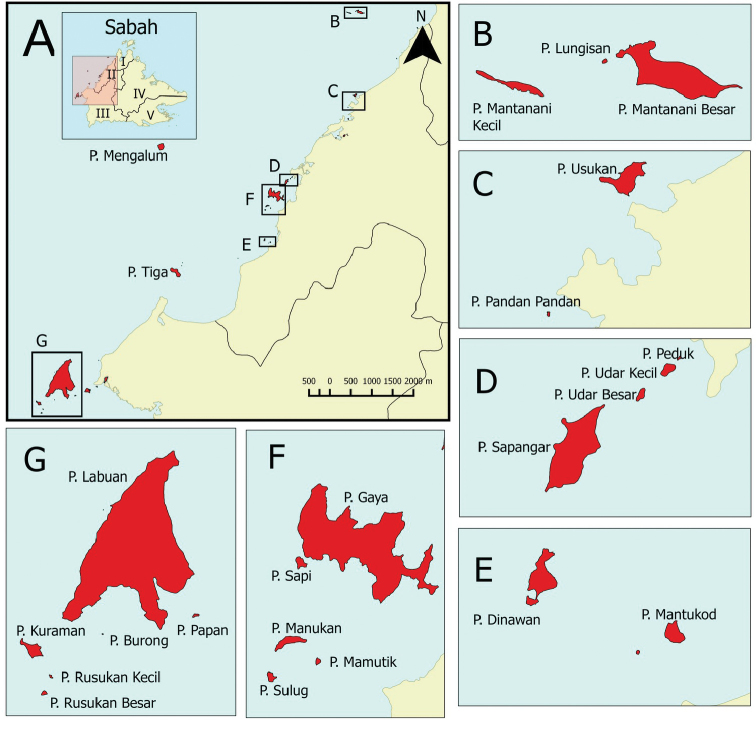
The 24 islands located off the Sabah west coast sampled for this study. **A** Sabah overview map with five divisions shown, namely (I) Kudat division, (II) West Coast division, (III) Interior division, (IV) Sandakan division, (V) Tawau division; Pulau Mengalum and Pulau Tiga shown in enlarged Sabah west coast **B** Mantanani island group **C** Pulau Usukan and Pulau Pandan Pandan **D** Sapangar Bay island group **E** Pulau Dinawan and Pulau Mantukod **F** Tunku Abdul Rahman Park **G** Pulau Labuan, Pulau Papan, Pulau Burong and Labuan Marine Park, which includes Pulau Kuraman, Pulau Rusukan Kecil and Pulau Rusukan Besar.

In view of these, a systematic sampling for land snails on 24 west coast islands was conducted and the data compiled together with land snail inventory data from literature and previously collected specimens deposited in the BORNEENSIS collection at Universiti Malaysia Sabah. Although only about half of the total number of islands on this part of Sabah were involved, the selection included the entire geographical extent of islands off the west coast of Sabah (see Figure 1). The aim of this paper is to provide a comprehensive and updated checklist of land snails by consolidating data from literature and database, including photographs of all species, in order to present a baseline species inventory for future island land snail studies. Interpretation of species diversity pattern in term of island biogeography will be discussed in upcoming analytical papers.

## Materials and methods

### Site of study

The Malay language term for ‘island’- *pulau* - will henceforth be used as many of the formally gazetted island names are in the local language. Land snails from 24 islands located off the Sabah west coast were investigated (see Figure 1). The sizes of these islands range from 0.005 km² (Pulau Peduk) to 87 km² (Pulau Labuan) (see Table 1). The distance between these islands and the mainland ranges from 0.25 km to 60 km. Eleven of the selected islands are in the three gazetted parks, namely Tunku Abdul Rahman Park, Pulau Tiga Park and Labuan Marine Park.

**Table 1.** Coordinates, areas (km²), numbers of standard plots, and numbers of existing records from BORNEENSIS for the 24 sampled islands.

Pulau (*Island*)	Latitude (°) / Longitude (°)	Area (km²)	Number of standard plots	Number of existing records from BORNEENSIS
1. Pulau Burong	5.2376, 115.1910	0.0108	1	7
2. Pulau Dinawan	5.8471, 115.9906	0.2603	5	51
3. Pulau Gaya	6.0175, 116.0315	14.5061	15	176
4. Pulau Kuraman	5.2245, 115.1315	1.5834	6	108
5. Pulau Labuan	5.3110, 115.2191	90.6642	11	147
6. Pulau Lungisan	6.7152, 116.3359	0.0132	6	11
7. Pulau Mamutik	5.9665, 116.0136	0.0563	3	46
8. Pulau Mantanani Besar	6.7119, 116.3529	2.0554	9	76
9. Pulau Mantanani Kecil	6.7092, 116.3129	0.2738	7	27
10. Pulau Mantukod	5.8378, 116.0129	0.0965	3	13
11. Pulau Manukan	5.9752, 116.0012	0.4196	8	52
12. Pulau Mengalum	6.2001, 115.5967	5.1640	7	112
13. Pulau Pandan Pandan	6.3508, 116.3087	0.0144	2	7
14. Pulau Papan	5.2537, 115.2706	0.1291	4	43
15. Pulau Peduk	6.0873, 116.0963	0.0049	2	11
16. Pulau Rusukan Besar	5.1873, 115.1413	0.1529	4	78
17. Pulau Rusukan Kecil	5.2015, 115.1470	0.0626	3	39
18. Pulau Sapi	6.0095, 116.0060	0.1856	2	18
19. Pulau Sapangar	6.0675, 116.0738	1.3225	7	76
20. Pulau Sulug	5.9598, 115.9932	0.1250	3	25
21. Pulau Tiga	5.7234, 115.6522	7.1638	11	257
22. Pulau Udar Besar	6.0794, 116.0881	0.0386	3	18
23. Pulau Udar Kecil	6.0848, 116.0939	0.0642	3	21
24. Pulau Usukan	6.3963, 116.3349	0.8787	8	188

### Land snail sampling and sorting

Field work was carried out between December 2015 and November 2016 with standard sampling conducted within a 20m × 20m plot (Schilthuizen et al. 2013). The number of plots varied from two to 14 depending on island size. Direct search for living land snails in their habitats such as vegetation, decaying logs and leaf litter on forest floor at each plot was carried out by two person-hours. Land snails encountered opportunistically along these trails were also collected. In addition, 5 liters of leaf litter and top soil were collected at each plot. The soil samples were then oven-dried for three days and subjected to a series of sieves. The empty shells were subsequently sorted out from the soil samples under a dissecting microscope.

### Land snail identification and accession

All the specimens were preserved and identified to species level based on shell characteristics by referring to the manuscript of *Field Guide to Land Snails of Sabah* (Vermeulen et al. in prep), Vermeulen et al. (2015), literature with original species descriptions, and the BORNEENSIS collection at the Institute for Tropical Biology and Conservation at Universiti Malaysia Sabah. After identification, specimens were then catalogued in the database, labelled and stored in the BORNEENSIS collection.

### Species checklist compilation

This checklist enumerates the land snail fauna sampled and recorded from the west coast islands between 2000 and 2016 based on literature, BORNEENSIS collections and recent systematic samplings (Table 1). Taxonomy classification of the species in this checklist followed the system used in the recent systematic paper on Sabah land snails (Vermeulen et al. 2015). The checklist comprised information about the type locality of the species, examined materials with accession number, species distribution in Sabah and general remarks for the species. For morphospecies that could not be assigned to an existing taxonomic name, a provisional species name was given (for example, sp. 1). Photographic images comprising four views of the representative specimen (i.e. apertural, lateral, dorsal and ventral views) for each species were presented.

### Type localities

The type locality of each species was written in the format of “country: state: district–location” if their exact location were known. For those without the exact location specified, a general description of the location (e.g. “Borneo”) was mentioned. “Not stated” was given to species with no locality indicated in their original description. For provisional species name, type locality was indicated as “not applicable”.

### Examined materials

For each species, the accession number of referenced specimens from the west coast islands was listed out. The abbreviation “BOR/MOL” indicated specimens from the BORNEENSIS collection while “V” referred to the private collection of Jaap J. Vermeulen of Leiden in the Netherlands. “Not seen” was mentioned for materials based solely on literature that could not be examined.

### Distribution in Sabah

The distribution of each species on the Sabah islands and mainland was compiled from the BORNEENSIS collection which currently houses more than 12,000 records. The distribution of each species was indicated in the format of “*Island*: [West]; [North]; [East]. *Mainland*:” Islands in Sabah were grouped into three categories: [West] for islands located within the West Coast Division and Interior Division; [North] for those located within Kudat Division, and [East] for ones situated within the Sandakan Division and Tawau Division. The distribution of the species on the mainland was according to five divisions: West Coast Division, Kudat Division, Sandakan Division, Tawau Division and Interior Division (Figure 1A).

### Remarks

Additional information such as species status as either endemic or introduced to Sabah or Borneo was stated for species well supported by literature. Conversely, those with no information from the literature were not mentioned. Other relevant notes for the species were also provided.

## Results and discussion

The checklist reported a total of 67 land snail species belonging to 37 genera and 19 families, of which 18 species were Caenogastropoda, four Neritimorpha and 45 Pulmonata (See supplementary file 1). The family Ariophantidae was the most species-rich family found on west coast islands, with eleven species (17.6%) recorded. This was followed by the family Cyclophoridae (nine species, 13.2%) and family Euconulidae (six species, 8.8%). Microsnails (size less than 5mm) accounted for about 47% of the total number of species. Among the 67 species, 19 were endemic, and six were introduced species. Species that were widespread across west coast islands included *Kaliella scandens* 20 islands, *Pythia chrysostoma* 20 islands, *Leptopoma pellucidum* 18 islands, *Paropeas achatinaceum* 18 islands, *Allopeas gracile* 18 islands, and *Ptychopatula orcula* 18 islands. Among these species, *Leptopoma pellucidum* was the only Caenogastropod found widespread across the west coast islands.

Surprisingly, this study revealed a high number of species that were unique to one particular island. 23 land snail species were found only on one of the 24 west coast islands included in this study. These were *Elasmias globulosum*, *Acmella striata*, *Japonia balabacensis*, *Platyraphe bongaoensis*, and *Plectostoma jucundum* (Pulau Mantanani Besar); *Charopa* sp. “lissobasis”, *Everettia* sp. , *Microcystina* sp. 2, and *Kaliella barrakporensis* (Pulau Labuan); *Ganesella tigaensis*, *Pterocyclos tenuilabiatus*, *Georissa scalinella* and *Georissa saulae* (Pulau Tiga); *Ditropopsis imadatei*, *Ditropopsis koperbergi* and *Pterocyclos amabilis* (Pulau Gaya); *Acmella polita* and *Arinia* sp. (Pulau Rusukan Besar); *Diplommatina recta* and *Videna metcalfei* (Pulau Mengalum); *Charopa* sp. “jugalis” (Pulau Sapangar); *Microcystina* sp. 1 (Pulau Peduk); and *Truncatella marginata* (Pulau Burong). From all the listed species above, nine were island endemics as these species had not been reported from mainland Sabah previously (i.e. *Platyraphe bongaoensis*, *Japonia balabacensis*, *Everettia* sp., *Microcystina* sp. 1, *Microcystina* sp. 2, *Ganesella tigaensis*, *Elasmias globulosum*, *Diplommatina recta*, ﻿and *Plectostoma jucundum*). Most of these species were recorded from Pulau Labuan, Mantanani group, and also from all the gazetted marine parks: Pulau Tiga Marine Park, Tunku Abdul Rahman Park and Labuan Marine Park, indicating that marine parks as vital habitats for unique species of land snails.

Among the 24 west coast islands, Pulau Labuan had the highest species richness (see supplementary file 1) and the highest number of species unique to the island. This could probably be due to its larger size compared to other islands. The second largest island on the west coast, Pulau Gaya, ranked fourth in species richness. Pulau Tiga and Pulau Mantanani Besar also recorded remarkable species richness, with 25 species from Pulau Tiga and 24 from Pulau Mantanani Besar. These two islands differed from other west coast islands in having limestone outcrops (in Pulau Mantanani Besar) and mud volcanoes (in Pulau Tiga), which potentially housed more diverse habitats leading to the existence of more land snail species.

Nevertheless, many species from Pulau Labuan in the 19^th^ century (Pfeiffer 1863; Adams 1865; Godwin-Austen 1891; Fulton 1896) were not found in this study. This included *Dyakia hugonis* (Pfeiffer, 1863), *Pterocyclos labuanensis* (Pfeiffer, 1863), *Geotrochus labuanensis* (Pfeiffer, 1863), *Ganesella subflava* (Godwin-Austen, 1891), *Trachia pudica* (Godwin-Austen, 1891), and Nanina (Xesta) decrespignyi (Higgins, 1868). On the other hand, three species were added to the previous land snail list of the Mantanani Island group by Schilthuizen et al. (2013) – *Elasmias manilense*, *Macrochlamys tersa*, and *Nesopupa moreleti*. However, four other species were not encountered during recent surveys–*Japonia balabacensis*, *Gastrocopta recondita*, *Elasmias globulosum* and *Microcystina microrhynchus*. In addition, four species, including some Borneo-endemic species found in Pulau Tiga during 2000 and 2003 were not encountered again during recent surveys –*Georissa saulae*, *Georissa scalinella*, *Geotrochus conicoides* and *Pterocyclos tenuilabiatus*.

The decline of land snail species (especially endemics) on the west coast islands could be attributed to two reasons. First, the field sampling period coincided partly with El Nino which had caused six months of drought in Sabah between January 2016 and June 2016 and might have negatively influenced sampling effectiveness. Second, forested habitats on many islands have vanished due to rapid urban development and tourism activities, particularly in Pulau Labuan (Mohamad and Samion 2016). Many of the species from Pulau Labuan were described or recorded from limestone outcrops in the south-eastern part of the island, all of which had since been quarried away for the construction of Labuan Airport in the 1960s. Hence, there was a high possibility that limestone-restricted species from Pulau Labuan could have been extinct in local.

However, six introduced land snail species were encountered across the 24 west coast islands –*Subulina octona*, *Achatina fulica*, *Huttonella bicolor*, *Macrochlamys indica*, *Quantula striata* and *Bradybaena similaris*. A large quantity of *Macrochlamys indica* and *Quantula striata* were found only on Pulau Labuan and Pulau Papan with *Quantula striata* a new record for Sabah land snails. The impact of the invasion by these introduced species on island land snail communities remains unknown. Therefore, future research should focus on the effects of introduced species on native species.

Although only 24 out of the 500 islands in Sabah were included, this study has managed to document nearly 20% of the total land snail species in Sabah. West coast islands are significant as habitats for land snails, particularly for several endemic species. In view of this, islands within the Labuan Marine Park, Pulau Tiga Park, Tunku Abdul Rahman Park, Pulau Labuan and Mantanani Island group should be prioritised for land snail conservation. Particular attention should also be accorded to island endemic species such as *Plectostoma jucundum* (endemic to Pulau Mantanani Besar) and *Ganesella tigaensis* (endemic to Pulau Tiga). Biodiversity conservation plans are therefore advocated not only for marine parks, but also for other west coast islands in Sabah.

## Species checklist

### Clade: CAENOGASTROPODA Cox

#### FAMILY ASSIMINEIDAE

##### Acmella striata

Taxon classificationAnimaliaLittorinimorphaAssimineidae

Vermeulen, Liew & Schilthuizen, 2015

Figure 2A

###### Type locality.

“Malaysia: Sabah: Kudat–Balambangan Island, South end, Batu Sireh” (Vermeulen et al. 2015)

**Figure 2.**
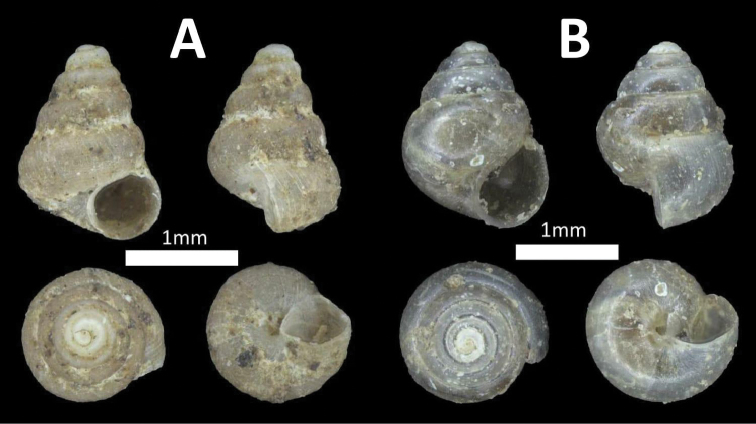
Family Assimineidae. **A** *Acmella striata* (BOR/MOL 7173) **B** *Acmella polita* (BOR/MOL 12243).

###### Examined materials.

*Pulau Mantanani Besar*: BOR/MOL 3726, BOR/MOL 7173.

###### Distribution in Sabah.

*Island*: [West] Pulau Mantanani Besar; [North] Pulau Balambangan. *Mainland*: Interior Division, Sandakan Division, and Tawau Division.

###### Remarks.

Widespread in Sabah.

##### Acmella polita

Taxon classificationAnimaliaLittorinimorphaAssimineidae

Von Moellendorff, 1887

Figure 2B

###### Type locality.

“Philippine: Luzon–Montalban near Manilla” (Von Moellendorff, 1887)

###### Examined materials.

*Pulau Rusukan Besar*: BOR/MOL 12237, BOR/MOL 12243.

###### Distribution in Sabah.

*Island*: [West] Pulau Rusukan Besar. *Mainland*: Interior Division, Sandakan Division and Tawau Division.

###### Remarks.

Widespread in Sabah. First record on an offshore island.

#### FAMILY CYCLOPHORIDAE

##### Pterocyclos tenuilabiatus

Taxon classificationAnimaliaArchitaenioglossaCyclophoridae

(Metcalfe, 1851)

Figure 3A

###### Type locality.

“Borneo” (Metcalfe, 1851)

**Figure 3.**
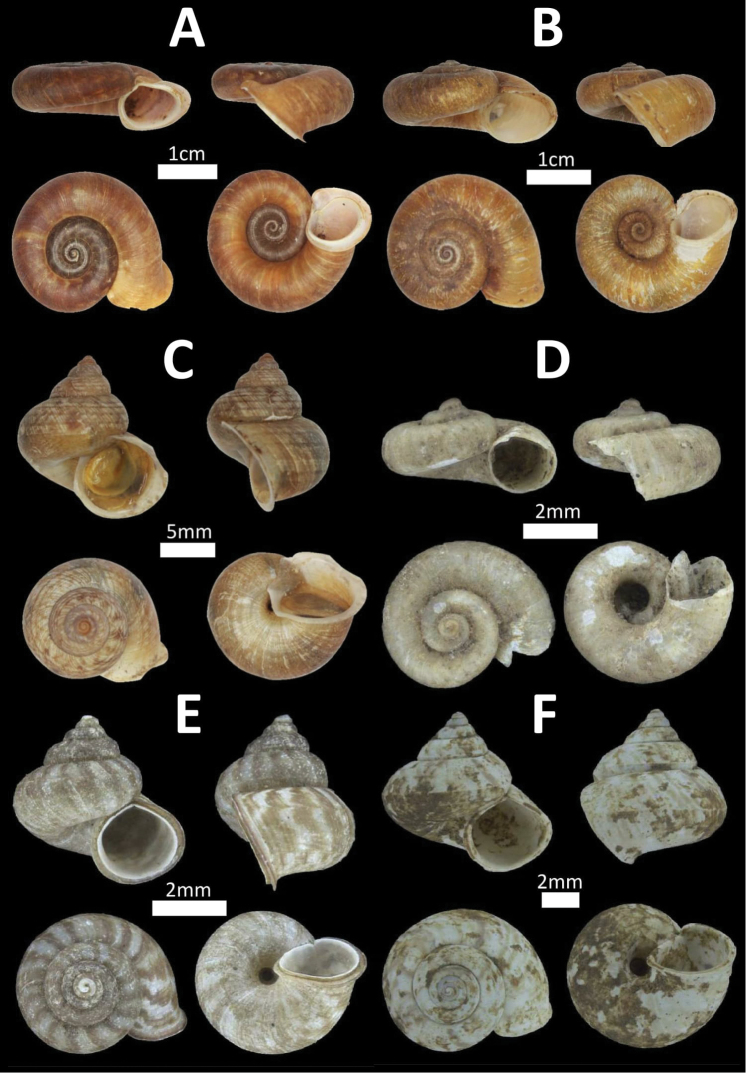
Family Cyclophoridae. **A** *Pterocyclos tenuilabiatus* (BOR/MOL 105) **B** *Pterocyclos amabilis* (BOR/MOL 8850) **C** *Leptopoma pellucidum* (BOR/MOL 6780) **D** *Platyraphe bongaoensis* (BOR/MOL 7185)*Broken aperture **E** *Japonia trilirata*/*kinabaluensis* species complex (BOR/MOL 12267) **F** *Japonia balabacensis* (BOR/MOL 3725).

###### Examined materials.

*Pulau Tiga*: BOR/MOL 105.

###### Distribution in Sabah.

*Island*: [West] Pulau Tiga. *Mainland*: West Coast Division, Interior Division, Sandakan Division and Tawau Division.

###### Remarks.

Endemic to Borneo. The examined sample was collected in 2000 and not found in the current surveys.

##### Pterocyclos amabilis

Taxon classificationAnimaliaArchitaenioglossaCyclophoridae

Fulton, 1905

Figure 3B

###### Type locality.

“N. Borneo” (Fulton, 1905)

###### Examined materials.

*Pulau Gaya*: BOR/MOL 8850, BOR/MOL 11118.

###### Distribution in Sabah.

*Island*: [West] Pulau Gaya. *Mainland*: West Coast Division and Interior Division.

###### Remarks.

Endemic to Sabah.

##### Leptopoma pellucidum

Taxon classificationAnimaliaArchitaenioglossaCyclophoridae

(Grateloup, 1840)

Figure 3C

###### Type locality.

“Philippines: Manilla” (Grateloup, 1839)

###### Examined materials.

*Pulau Mantanani Besar*: BOR/MOL 1562, BOR/MOL 1573, BOR/MOL 3723, BOR/MOL 3724, BOR/MOL 6008, BOR/MOL 6009, BOR/MOL 6011, BOR/MOL 6012, BOR/MOL 6013, BOR/MOL 6699, BOR/MOL 6700, BOR/MOL 6701, BOR/MOL 6702, BOR/MOL 6704, BOR/MOL 7187. *Pulau Mantanani Kecil*: BOR/MOL 3727, BOR/MOL 3728, BOR/MOL 3787. *Pulau Mengalum*: BOR/MOL 6064, BOR/MOL 6065, BOR/MOL 6066, BOR/MOL 6162, BOR/MOL 6168, BOR/MOL 6178, BOR/MOL 8733, BOR/MOL 8737, BOR/MOL 8738, *Pulau Tiga*: BOR/MOL 8817, BOR/MOL 9720, BOR/MOL 9722, BOR/MOL 9726, BOR/MOL 9727, BOR/MOL 9728. *Pulau Gaya*: BOR/MOL 6301, BOR/MOL 6303, BOR/MOL 8816, BOR/MOL 8851, BOR/MOL 9444, BOR/MOL 9445, BOR/MOL 9446, BOR/MOL 9447, BOR/MOL 9449, BOR/MOL 9450, BOR/MOL 9451. *Pulau Sapangar*: BOR/MOL 6784, BOR/MOL 6786, BOR/MOL 6791, BOR/MOL 6792, BOR/MOL 6794, BOR/MOL 6796, BOR/MOL 6777, BOR/MOL 6780, BOR/MOL 6782, BOR/MOL 12000, BOR/MOL 12006, BOR/MOL 12013, BOR/MOL 12014, BOR/MOL 12015. *Pulau Udar Besar*: BOR/MOL 6802, BOR/MOL 6803, BOR/MOL 6804. *Pulau Udar Kecil*: BOR/MOL 7151, BOR/MOL 7152, BOR/MOL 7153, BOR/MOL 7154, BOR/MOL 7155, BOR/MOL 7156, BOR/MOL 10379. *Pulau Sapi*: BOR/MOL 6668, BOR/MOL 6669, BOR/MOL 6670, BOR/MOL 7933, BOR/MOL 8523, BOR/MOL 8524. *Pulau Mamutik*: BOR/MOL 6691, BOR/MOL 6693, BOR/MOL 6694, BOR/MOL 6695, BOR/MOL 6764, BOR/MOL 6767, BOR/MOL 10000, BOR/MOL 10009. *Pulau Manukan*: BOR/MOL 6744, BOR/MOL 6745, BOR/MOL 6746, BOR/MOL 6747, BOR/MOL 6749, BOR/MOL 6752, BOR/MOL 6755. *Pulau Sulug*: BOR/MOL 6769, BOR/MOL 6770, BOR/MOL 6771, BOR/MOL 7932, BOR/MOL 10338, BOR/MOL 10346. *Pulau Usukan*: BOR/MOL 7886, BOR/MOL 7887, BOR/MOL 7888, BOR/MOL 7881, BOR/MOL 7882, BOR/MOL 12024, BOR/MOL 12025, BOR/MOL 12027, BOR/MOL 12039, BOR/MOL 12040, BOR/MOL 12475. *Pulau Labuan*: BOR/MOL 7913, BOR/MOL 7917, BOR/MOL 7904, BOR/MOL 7906, BOR/MOL 7907, BOR/MOL 7910, BOR/MOL 8590, BOR/MOL 8795, BOR/MOL 12168, BOR/MOL 12184. *Pulau Dinawan*: BOR/MOL 7678, BOR/MOL 7679, BOR/MOL 7680, BOR/MOL 7681, BOR/MOL 7683, BOR/MOL 7693, BOR/MOL 7694, BOR/MOL 8909. *Pulau Rusukan Kecil*: BOR/MOL 8552, BOR/MOL 8555, BOR/MOL 8556, BOR/MOL 8557, BOR/MOL 8558, BOR/MOL 8605, BOR/MOL 8539, BOR/MOL 8540, BOR/MOL 8541, BOR/MOL 8542, BOR/MOL 8543, BOR/MOL 8544, BOR/MOL 8545, BOR/MOL 8546, BOR/MOL 8550. *Pulau Rusukan Besar*: BOR/MOL 8559, BOR/MOL 8560, BOR/MOL 8574, BOR/MOL 8575, BOR/MOL 8576, BOR/MOL 8583, BOR/MOL 8584, BOR/MOL 8585, BOR/MOL 8588, BOR/MOL 8589, BOR/MOL 12234, BOR/MOL 12247, BOR/MOL 12265, BOR/MOL 12271. *Pulau Kuraman*: BOR/MOL 8614, BOR/MOL 8621, BOR/MOL 8622, BOR/MOL 8626, BOR/MOL 8631, BOR/MOL 8635, BOR/MOL 8638, BOR/MOL BOR/MOL 12099, BOR/MOL 12108, BOR/MOL 12120, BOR/MOL 12134, BOR/MOL 12144.

###### Distribution in Sabah.

*Island*: [West] Tunku Abdul Rahman Park, Pulau Mengalum, Pulau Tiga, Pulau Sapangar, Pulau Udar Kecil, Pulau Udar Besar, Pulau Usukan, Pulau Labuan, Labuan Marine Park; [North] Pulau Banggi, Pulau Balambangan; [East] Pulau Bod Gaya. *Mainland*: Kudat Division, West Coast Division, Interior Division, Sandakan Division and Tawau Division.

###### Remarks.

Widespread in Sabah.

##### Platyraphe bongaoensis

Taxon classificationAnimaliaArchitaenioglossaCyclophoridae

(E.A. Smith, 1894)

Figure 3D

###### Type locality.

“Philippines: Sulu Archipelago: Tawi-Tawi–Bongao island” (E.A. Smith, 1894).

###### Examined materials.

*Pulau Mantanani Besar*: BOR/MOL 3722, BOR/MOL 7169, BOR/MOL 7185, BOR/MOL 7201.

###### Distribution in Sabah.

*Island*: [West] Pulau Mantanani Besar; [North] Pulau Balambangan. *Mainland*: No record.

###### Remarks.

Only found in Pulau Mantanani Besar and Pulau Balambangan, which are close to the Palawan archipelago in the Philippines.

##### Japonia trilirata /kinabaluensis species complex

Taxon classificationAnimaliaArchitaenioglossaCyclophoridae

Figure 3E

###### Type locality.

*Japonia kinabaluensis* “Malaysia: Sabah: Ranau–Mt. Kinabalu” (Smith, 1895); *Japonia trilirata* “Malaysia: Sabah: Labuan” (Pfeiffer, 1852)

###### Examined materials.

*Pulau Tiga*: BOR/MOL 201, BOR/MOL 202, BOR/MOL 2758, BOR/MOL 8518, BOR/MOL 6606, BOR/MOL 6595, BOR/MOL 8425, BOR/MOL 8431, BOR/MOL 11079, BOR/MOL 11087, BOR/MOL 11091, BOR/MOL 11093, BOR/MOL 11107, BOR/MOL 11109, BOR/MOL 11110. *Pulau Gaya*: BOR/MOL 8461, BOR/MOL 8475, BOR/MOL 8477, BOR/MOL 8449, BOR/MOL 8495, BOR/MOL 8502, BOR/MOL 9448, BOR/MOL 10988, BOR/MOL 10359, BOR/MOL 10369. *Pulau Rusukan Besar*: BOR/MOL 8577, BOR/MOL 8578, BOR/MOL 8579, BOR/MOL 8586, BOR/MOL 12227, BOR/MOL 12242, BOR/MOL 12246, BOR/MOL 12255, BOR/MOL 12260, BOR/MOL 12266, BOR/MOL 12267, BOR/MOL 12270. *Pulau Kuraman*: BOR/MOL 8615, BOR/MOL 8632, BOR/MOL 8636, BOR/MOL 8639, BOR/MOL 8644, BOR/MOL 8647, BOR/MOL 8656, BOR/MOL 12103, BOR/MOL 12105, BOR/MOL 12114, BOR/MOL 12126, BOR/MOL 12136, BOR/MOL 12139, BOR/MOL 12145. *Pulau Dinawan*: BOR/MOL 8922, BOR/MOL 8905, BOR/MOL 8915, BOR/MOL 8917. *Pulau Sapangar*: BOR/MOL 11991, BOR/MOL 11997, BOR/MOL 12011. *Pulau Labuan*: BOR/MOL 12194.

###### Distribution in Sabah.

*Island*: [West] Pulau Tiga, Pulau Gaya, Pulau Rusukan Besar, Pulau Kuraman, Pulau Dinawan, Pulau Sapangar, Pulau Labuan; [East] Pulau Bod Gaya, Pulau Bohey Dulang, Pulau Sebangkat, Pulau Selakan, Pulau Tetagan. *Mainland*: West Coast Division, Interior Division, Sandakan Division and Tawau Division.

###### Remarks.

Widespread in Sabah. The difference between *Japonia kinabaluensis* (E.A. Smith, 1895) and *Japonia trilirata* (Pfeiffer, 1852) is ambiguous as the shell shape and size of the two species are highly variable and intermediate shell forms have been found. Therefore, we considered *J. kinabaluensis* and *J. trilirata* as *Japonia trilirata*/*kinabaluensis* species complex.

##### Japonia balabacensis

Taxon classificationAnimaliaArchitaenioglossaCyclophoridae

(E.A. Smith, 1895)

Figure 3F

###### Type locality.

“Philippines: Palawan–Balabac Island” (E.A. Smith, 1895)

###### Examined materials.

*Pulau Mantanani Besar*: BOR/MOL 3725.

###### Distribution in Sabah.

*Island*: [West] Pulau Mantanani Besar; [North] Pulau Banggi, Pulau Balambangan. *Mainland*: No record.

###### Remarks.

Only found in Pulau Mantanani Besar, Pulau Balambangan and Pulau Banggi, which are close to the Palawan archipelago in the Philippines.

##### Japonia keppeli

Taxon classificationAnimaliaArchitaenioglossaCyclophoridae

(Godwin-Austen, 1889)

Figure 4A

###### Type locality.

“Malaysia: Sarawak: Miri–Batu Niah N.P” (Godwin-Austen, 1889)

**Figure 4.**
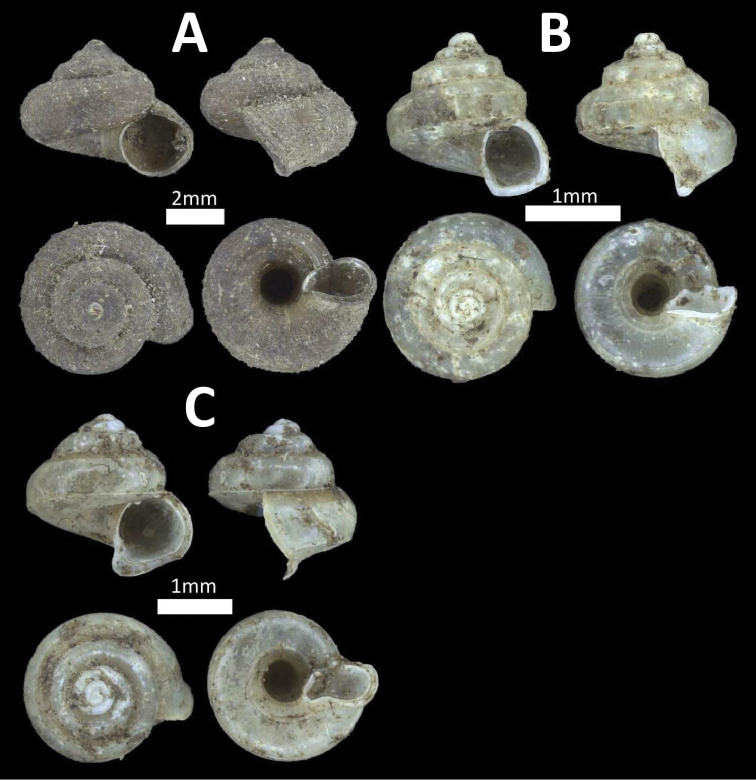
Family Cyclophoridae. **A** *Japonia keppeli* (BOR/MOL 10027) **B** *Ditropopsis koperbergi* (BOR/MOL 8455) **C** *Ditropopsis imadatei* (BOR/MOL 10358).

###### Examined materials.

*Pulau Gaya*: BOR/MOL 6681, BOR/MOL 6683, BOR/MOL 8466, BOR/MOL 8481, BOR/MOL 8489, BOR/MOL 8494, BOR/MOL 8501, BOR/MOL 10363, BOR/MOL 10368. *Pulau Sapi*: BOR/MOL 10027, BOR/MOL 10293. *Pulau Manukan*: BOR/MOL 10298, BOR/MOL 10302, BOR/MOL 10303, BOR/MOL 10307, BOR/MOL 10316, BOR/MOL 10318, BOR/MOL 10324. *Pulau Sulug*: BOR/MOL 10330, BOR/MOL 10332, BOR/MOL 10333, BOR/MOL 10339, BOR/MOL 10345.

###### Distribution in Sabah.

*Island*: [West] Pulau Gaya, Pulau Sapi, Pulau Manukan, Pulau Sulug. *Mainland*: Interior Division.

###### Remarks.

Endemic to Borneo.

##### Ditropopsis koperbergi

Taxon classificationAnimaliaArchitaenioglossaCyclophoridae

(Zilch, 1955)

Figure 4B

###### Type locality.

“Indonesia: Kalimantan: Landak” (Zilch, 1955)

###### Examined materials.

*Pulau Gaya*: BOR/MOL 8455, BOR/MOL 8446.

###### Distribution in Sabah.

*Island*: [West] Pulau Gaya. *Mainland*: Interior Division, West Coast Division and Sandakan Division.

###### Remarks.

Endemic to Borneo.

##### Ditropopsis imadatei

Taxon classificationAnimaliaArchitaenioglossaCyclophoridae

(Habe, 1965)

Figure 4C

###### Type locality.

“Brunei: Bandar Seri Begawan” (Habe, 1965)

###### Examined materials.

*Pulau Gaya*: BOR/MOL 8460, BOR/MOL 10358.

###### Distribution in Sabah.

*Island*: [West] Pulau Gaya. *Mainland*: Interior Division.

###### Remarks.

Endemic to Borneo.

#### FAMILY DIPLOMMATINIDAE

##### Diplommatina recta

Taxon classificationAnimaliaMesogastropodaDiplommatinidae

E.A. Smith, 1895

Figure 5A

###### Type locality.

“Malaysia: Sabah: Ranau–Mt. Kinabalu” (E.A. Smith, 1895)

**Figure 5.**
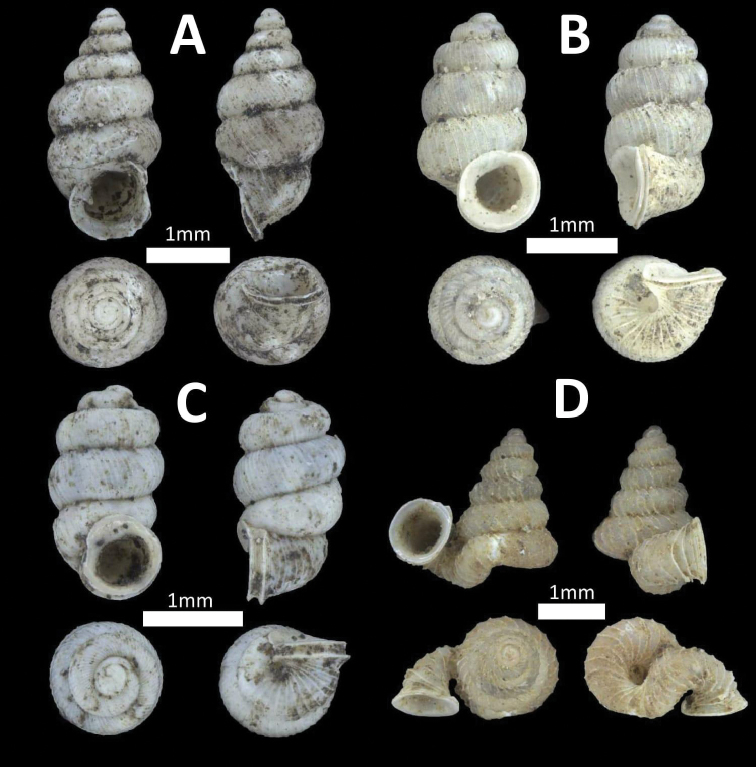
Family Diplommatinidae. **A** *Diplommatina recta* (BOR/MOL 12297) **B** *Arinia borneensis* (BOR/MOL 12053) **C** *Arinia* sp. (BOR/MOL 12252) **D** *Plectostoma jucundum* (BOR/MOL 7179).

###### Examined materials.

*Pulau Mengalum*: BOR/MOL 12297.

###### Distribution in Sabah.

*Island*: [West] Pulau Mengalum; [North] Pulau Balambangan. *Mainland*: No record.

###### Remarks.

There is no recent record of this species from the mainland. The only record on the mainland is in the original description of the species by Smith (1895).

##### Arinia borneensis

Taxon classificationAnimaliaMesogastropodaDiplommatinidae

E.A. Smith, 1894

Figure 5B

###### Type locality.

“Malaysia: Sabah: Sandakan–Gomantong” (E.A. Smith, 1894b)

###### Examined materials.

*Pulau Mantanani Besar*: BOR/MOL 3721, BOR/MOL 7172, BOR/MOL 7177, BOR/MOL 7183. *Pulau Mantanani Kecil*: BOR/MOL 3729, BOR/MOL 7196. *Pulau Lungisan*: BOR/MOL 3742. *Pulau Tiga*: BOR/MOL 6598, BOR/MOL 11100. *Pulau Usukan*: BOR/MOL 12026, BOR/MOL 12028, BOR/MOL 12037, BOR/MOL 12048, BOR/MOL 12051, BOR/MOL 12053. *Pulau Kuraman*: BOR/MOL 12102, BOR/MOL 12107.

###### Distribution in Sabah.

*Island*: [West] Mantanani group, Pulau Tiga, Pulau Usukan, Pulau Kuraman. *Mainland*: Kudat Division, Sandakan Division and Tawau Division.

###### Remarks.

Endemic and widespread in Sabah.

##### Arinia

Taxon classificationAnimaliaMesogastropodaDiplommatinidae

sp.

Figure 5C

###### Type locality.

Not applicable.

###### Examined materials.

*Pulau Rusukan Besar*: BOR/MOL 12252.

###### Distribution in Sabah.

*Island*: [West] Pulau Rusukan Besar. *Mainland*: No record.

###### Remarks.

Unknown status. Does not match with any other *Arinia* species in Borneo treated in the revision by Vermeulen (1996).

##### Plectostoma jucundum

Taxon classificationAnimaliaMesogastropodaDiplommatinidae

(E.A. Smith, 1893)

Figure 5D

###### Type locality.

“Malaysia: Sabah: Kota Belud–Mantanani Island” (E.A. Smith, 1892)

###### Examined materials.

*Pulau Mantanani Besar*: BOR/MOL 3720, BOR/MOL 5601, BOR/MOL 7179.

###### Distribution in Sabah.

*Island*: [West] Pulau Mantanani Besar. *Mainland*: No record.

###### Remarks.

Endemic to Pulau Mantanani Besar. Critically endangered as this species is only found in one locality and is threatened by habitat loss (Schilthuizen and Vermeulen 2004a).

##### Diplommatina

Taxon classificationAnimaliaMesogastropodaDiplommatinidae

sp.

###### Type locality.

Not applicable.

###### Examined materials.

Pulau Sapangar: BOR/MOL 11993.

###### Distribution in Sabah.

*Island*: [West] Pulau Sapangar. *Mainland*: No record.

###### Remarks.

Unable to identify because the only shell found was juvenile.

#### FAMILY TRUNCATELLIDAE

##### Truncatella guerinii

Taxon classificationAnimaliaLittorinimorphaTruncatellidae

(Villa & Villa, 1841)

Figure 6A

###### Type locality.

“France: Reunion” (Villa & Villa, 1841)

**Figure 6.**
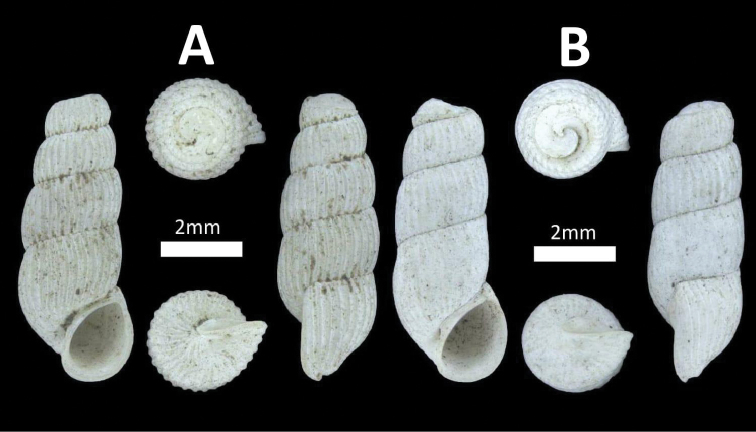
Family Truncatellidae. **A** *Truncatella guerinii* (BOR/MOL 12333) **B** *Truncatella marginata* (BOR/MOL 12445).

###### Examined materials.

*Pulau Mantanani Kecil*: BOR/MOL 3731. *Pulau Peduk*: BOR/MOL 10348. *Pulau Burong*: BOR/MOL 12333.

###### Distribution in Sabah.

*Island*: [West] Pulau Mantanani Kecil, Pulau Peduk, Pulau Burong; [North] Pulau Banggi, Pulau Balambangan. *Mainland*: West Coast Division.

###### Remarks.

Unknown status. Relatively scarce information about the distribution in Sabah as its habitat at coastal areas has rarely been surveyed. Presumably widespread along coastal areas since this species was known from across the Indo-pacific region (Clench and Turner 1948).

##### Truncatella marginata

Taxon classificationAnimaliaLittorinimorphaTruncatellidae

Küster, 1855

Figure 6B

###### Type locality.

“Malaysia: Sabah: Labuan” (Küster, 1855)

###### Examined materials.

*Pulau Burong*: BOR/MOL 12445.

###### Distribution in Sabah.

*Island*: [West] Pulau Burong. *Mainland*: No record.

###### Remarks.

Unknown status. Scarce information about the distribution in Sabah as its habitat at coastal area has rarely been surveyed.

### Unranked clade: NERITIMORPHA

#### FAMILY HELICINIDAE

##### Aphanoconia usukanensis

Taxon classificationAnimaliaLittorinimorphaTruncatellidae

(Godwin-Austen, 1889)

Figure 7

###### Type locality.

“Malaysia: Sabah: Kota Belud–Usukan island” (Godwin-Austen 1889)

**Figure 7.**
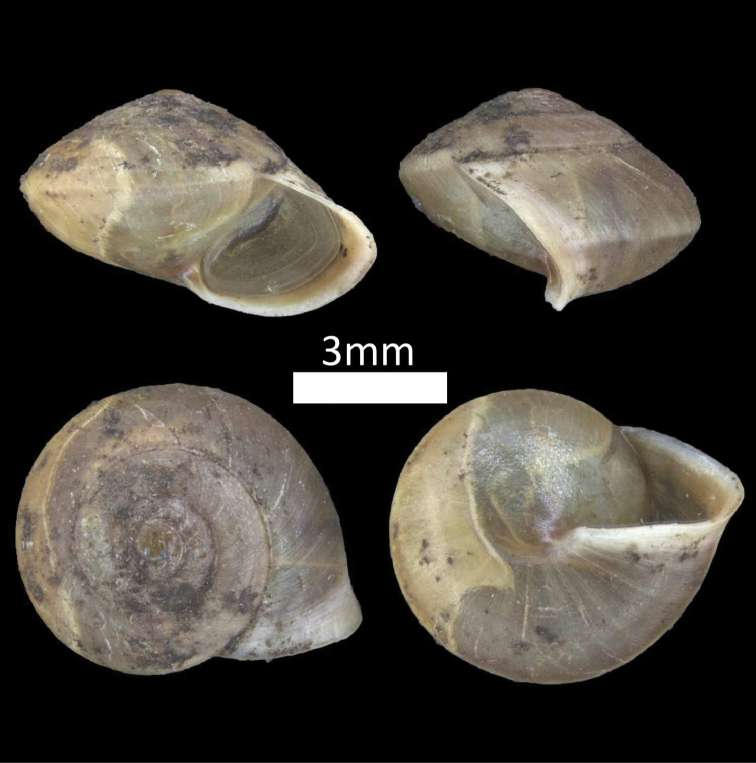
Family Helicinidae. *Aphanoconia usukanensis* (BOR/MOL 7200).

###### Examined materials.

*Pulau Mantanani Besar*: BOR/MOL 3719, BOR/MOL 6703, BOR/MOL 7164, BOR/MOL 7200. *Pulau Mantanani Kecil*: BOR/MOL 3730, BOR/MOL 3755. *Pulau Lungisan*: BOR/MOL 3743. *Pulau Mengalum*: BOR/MOL 6067, BOR/MOL 6163, BOR/MOL 6170, BOR/MOL 6179, BOR/MOL 8730, BOR/MOL 8734, BOR/MOL 8735, BOR/MOL 8736, BOR/MOL 8858, BOR/MOL 8859, BOR/MOL 8860, BOR/MOL 8863, BOR/MOL 9993, BOR/MOL 9996, BOR/MOL 9997, BOR/MOL 12345, BOR/MOL 12279, BOR/MOL 12288, BOR/MOL 12296, BOR/MOL 12302, BOR/MOL 12312, BOR/MOL 12315, BOR/MOL 12320. *Pulau Mamutik*: BOR/MOL 6692, BOR/MOL 6696, BOR/MOL 6697, BOR/MOL 6698, BOR/MOL 6765, BOR/MOL 6768, BOR/MOL 10008, BOR/MOL 10014, BOR/MOL 10023, BOR/MOL 10025. *Pulau Manukan*: BOR/MOL 6750, BOR/MOL 6751, BOR/MOL 6753, BOR/MOL 6754, BOR/MOL 6756, BOR/MOL 6761, BOR/MOL 6762, BOR/MOL 6772, BOR/MOL 10301, BOR/MOL 10309. *Pulau Usukan*: BOR/MOL 7884, BOR/MOL 7885, BOR/MOL 7893, BOR/MOL 7895, BOR/MOL 7898, BOR/MOL 7883, BOR/MOL 12477. *Pulau Dinawan*: BOR/MOL 7685, BOR/MOL 7692, BOR/MOL 8902, BOR/MOL 8911.

###### Distribution in Sabah.

*Island*: [West] Mantanani group, Pulau Mengalum, Pulau Mamutik, Pulau Manukan, Pulau Usukan, Pulau Dinawan; [North] Pulau Banggi, Pulau Balambangan; [East] Pulau Bohey Dulang, Pulau Sebangkat, Pulau Selakan, Pulau Tetagan, Pulau Mantabuan, Pulau Sibuan, Pulau Maiga. *Mainland*: Sandakan Division, and Tawau Division.

###### Remarks.

Endemic and widespread in Sabah.

#### FAMILY HYDROCENIDAE

##### Georissa saulae

Taxon classificationAnimaliaCycloneritimorphaHydrocenidae

(van Benthem Jutting, 1966)

Figure 8A

###### Type locality.

“Malaysia: Sabah: Keningau–Lian Cave” (van Benthem Jutting 1966)

**Figure 8.**
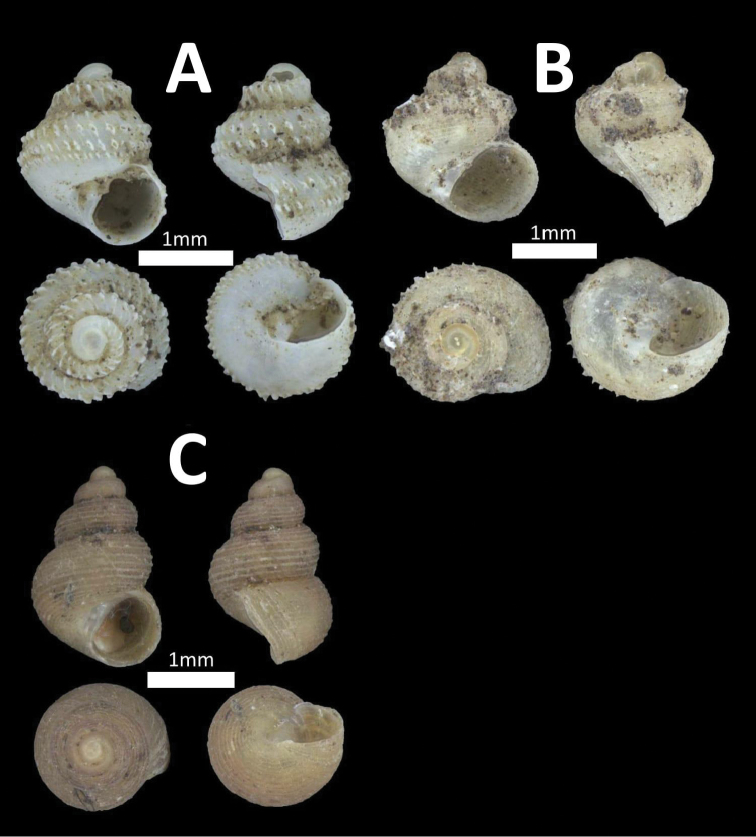
FAMILY HYDROCENIDAE. **A** *Georissa saulae* (BOR/MOL 27) **B** *Georissa scalinella* (BOR/MOL 41) **C** *Georissa williamsi* (BOR/MOL 7174).

###### Examined materials.

*Pulau Tiga*: BOR/MOL 27.

###### Distribution in Sabah.

*Island*: [West] Pulau Tiga. *Mainland*: West Coast Division and Interior Division.

###### Remarks.

Endemic to Sabah. The examined sample was collected in 2000 and not found in the current survey.

##### Georissa scalinella

Taxon classificationAnimaliaCycloneritimorphaHydrocenidae

(van Benthem Jutting, 1966)

Figure 8B

###### Type locality.

“Malaysia: Sabah: Lahad Datu–Lahad Batu Caves on Teck Guan Estate” (van Benthem Jutting 1966).

###### Examined materials.

*Pulau Tiga*: BOR/MOL 41.

###### Distribution in Sabah.

*Island*: [West] Pulau Tiga. *Mainland*: Interior Division, Sandakan Division, and Tawau Division.

###### Remarks.

Endemic to Sabah. The examined sample was collected in 2000 and not found in the current survey.

##### Georissa williamsi

Taxon classificationAnimaliaCycloneritimorphaHydrocenidae

Godwin-Austen, 1889

Figure 8C

###### Type locality.

“Borneo” (Godwin-Austen 1889)

###### Examined materials.

*Pulau Mantanani Besar*: BOR/MOL 3718, BOR/MOL 7161, BOR/MOL 7174. *Pulau Lungisan*: BOR/MOL 3744.

###### Distribution in Sabah.

*Island*: [West] Mantanani Island group. *Mainland*: West Coast Division and Sandakan Division.

###### Remarks.

Widespread in Sabah mainland.

### Clade: PULMONATA Cuvier

#### FAMILY ACHATINELLIDAE

##### Elasmias globulosum

Taxon classificationAnimaliaStylommatophoraAchatinellidae

Quoi & Gaimard ex Zilch, 1962

###### Type locality.

“Philippines: Mindanao” (Quoi & Gaimard ex Zilch 1962)

###### Examined materials.

Pulau Mantanani Besar: BOR/MOL 3717 (Not seen).

###### Distribution in Sabah.

*Island*: [West] Pulau Mantanani Besar; [North] Pulau Balambangan. *Mainland*: No record.

###### Remarks.

Only found in Pulau Mantanani Besar and Pulau Balambangan, which are close to the Palawan archipelago in the Philippines. This species is reported in Schilthuizen et al. (2013) but the specimen could not be found in the BORNEENSIS collection.

##### Elasmias manilense

Taxon classificationAnimaliaStylommatophoraAchatinellidae

(Dohrn, 1863)

Figure 9A

###### Type locality.

“Philippines: Luzon– near Manila” (Dohrn 1863)

**Figure 9.**
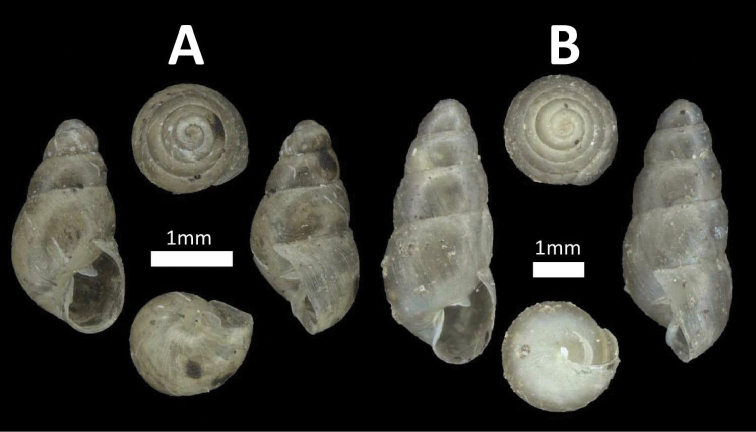
Family Achatinellidae. **A** *Elasmias manilense* (BOR/MOL 12356) **B** *Tornatellinops moluccana* (BOR/MOL 11974).

###### Examined materials.

*Pulau Mantanani Besar*: BOR/MOL 7163. *Pulau Gaya*: BOR/MOL 8470. *Pulau Rusukan Kecil*: BOR/MOL 8554, BOR/MOL 8607, BOR/MOL 8549, BOR/MOL 8551, BOR/MOL 12356. *Pulau Rusukan Besar*: BOR/MOL 8562, BOR/MOL 8567, BOR/MOL 8568, BOR/MOL 8582, BOR/MOL 12228, BOR/MOL 12241, BOR/MOL 12249, BOR/MOL 12254. *Pulau Kuraman*: BOR/MOL 8649, BOR/MOL 8651, BOR/MOL 12109.

###### Disribution in Sabah.

*Island*: [West] Pulau Mantanani Besar, Pulau Gaya, Labuan Marine Park; [East] Pulau Bohey Dulang, Pulau Mantabuan, Pulau Sibuan. *Mainland*: No record.

###### Remarks.

Widespread in Sabah on islands.

##### Tornatellinops moluccana

Taxon classificationAnimaliaStylommatophoraAchatinellidae

(Boettger, 1891)

Figure 9B

###### Type locality.

“Indonesia: Maluku–Ambon” (Boettger 1891)

###### Examined materials.

*Pulau Dinawan*: BOR/MOL 8912. *Pulau Udar Besar*: BOR/MOL 11075, BOR/MOL 11076. *Pulau Sapangar*: BOR/MOL 11974. *Pulau Gaya*: BOR/MOL 8470. *Pulau Rusukan Besar*: BOR/MOL 12228, BOR/MOL 12241, BOR/MOL 12542.

###### Distribution in Sabah.

*Island*: [West] Pulau Dinawan, Pulau Udar Besar, Pulau Sapangar, Pulau Gaya, Pulau Rusukan Besar. *Mainland*: Tawau Division.

###### Remarks.

Rather widespread in Sabah.

#### FAMILY ACHATINIDAE

##### Achatina fulica

Taxon classificationAnimaliaStylommatophoraAchatinidae

(Bowdich, 1822)

Figure 10

###### Type locality.

Not stated.

###### Examined materials.

*Pulau Mantanani Besar*: BOR/MOL 1824, BOR/MOL 3716, BOR/MOL 3756, BOR/MOL 7191. *Pulau Lungisan*: BOR/MOL 3745. *Pulau Labuan*: BOR/MOL 6592, BOR/MOL 7901, BOR/MOL 7903, BOR/MOL 7926, BOR/MOL 7927, BOR/MOL 8814, BOR/MOL 12183, BOR/MOL 12200, BOR/MOL 12214, BOR/MOL 12224, BOR/MOL 12338, BOR/MOL 12341, BOR/MOL 12344, BOR/MOL 12446, BOR/MOL 12482. *Pulau Dinawan*: BOR/MOL 8923. *Pulau Usukan*: BOR/MOL 12022, BOR/MOL 12023, BOR/MOL 12079, BOR/MOL 12080, BOR/MOL 12081, BOR/MOL 12339, BOR/MOL 12340, BOR/MOL 12480. *Pulau Papan*: BOR/MOL 12066.

**Figure 10.**
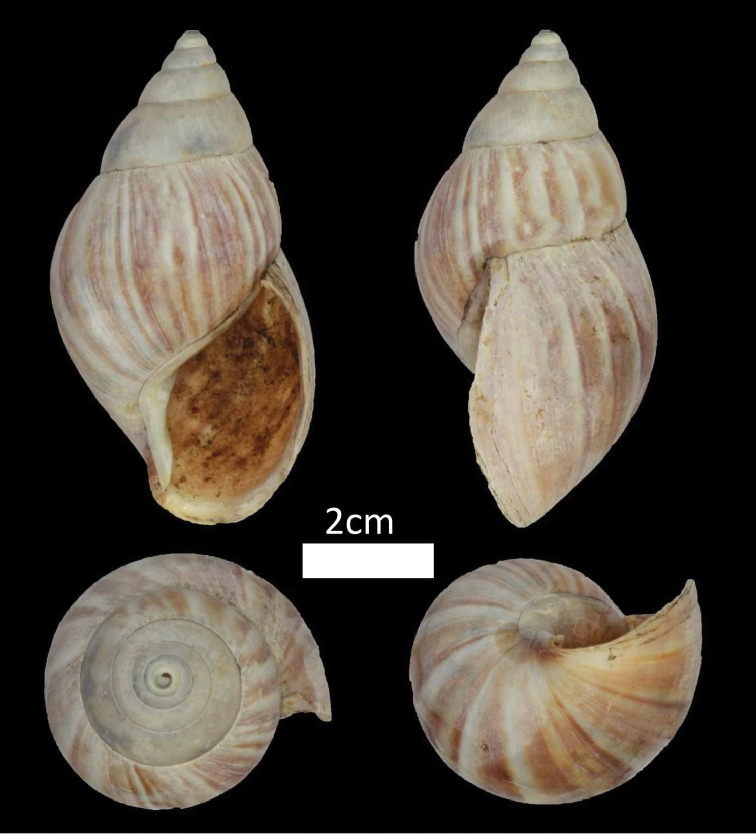
Family Achatinidae. *Achatina fulica* (BOR/MOL 12339).

###### Distribution in Sabah.

*Island*:[West] Pulau Mantanani Besar, Pulau Lungisan, Pulau Labuan, Pulau Dinawan, Pulau Usukan, Pulau Papan; [North] Pulau Banggi, Pulau Balambangan. *Mainland*: Kudat Division, West Coast Division, Interior Division, Sandakan Division, and Tawau Division.

###### Remarks.

Introduced and widespread in Sabah.

#### FAMILY ARIOPHANTIDAE

##### Hemiplecta humphreysiana

Taxon classificationAnimaliaStylommatophoraAriophantidae

(Lea, 1841)

Figure 11A

###### Type locality.

“India: Puducherry: Pondicherry” (Lea 1841)

**Figure 11.**
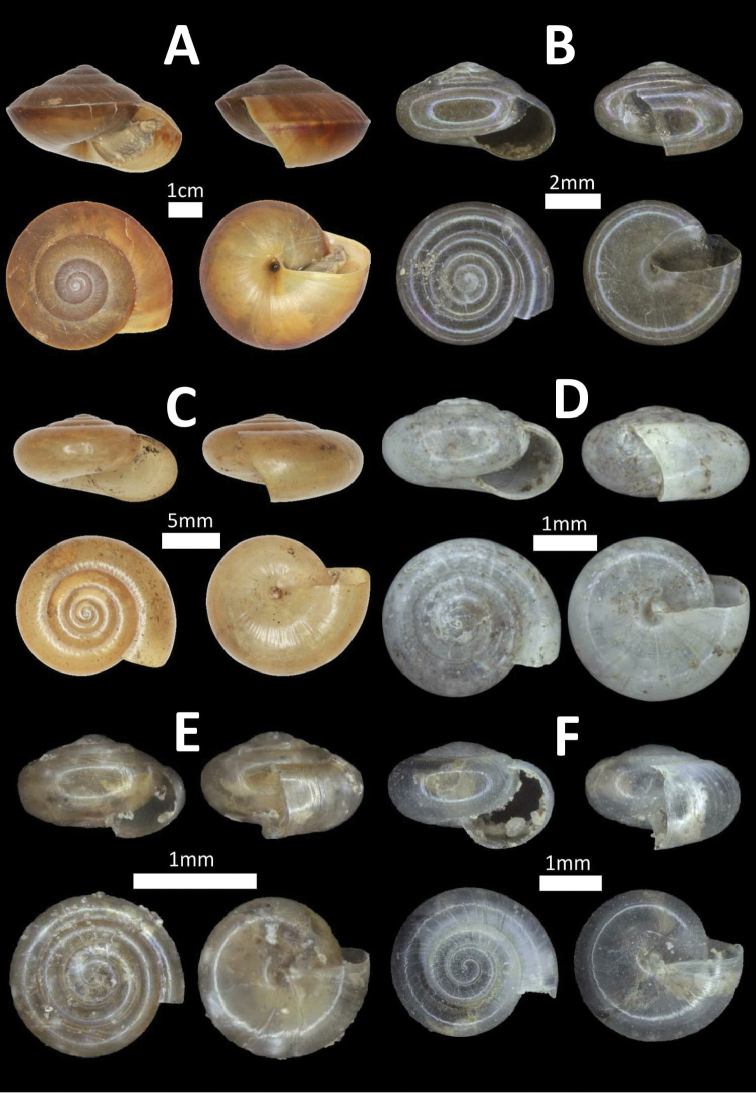
FAMILY ARIOPHANTIDAE. **A** *Hemiplecta humphreysiana* (BOR/MOL 7870) **B** *Macrochlamys tersa* (BOR/MOL 10299) **C** *Macrochlamys indica* (BOR/MOL 12490) **D** *Microcystina striatula* (BOR/MOL 10310) **E** *Microcystina sinica* (BOR/MOL 12047) **F** *Microcystina microrhynchus* (BOR/MOL 11982).

###### Examined materials.

*Pulau Tiga*: BOR/MOL 998, BOR/MOL 1008, BOR/MOL 1009, BOR/MOL 1389, BOR/MOL 4262, BOR/MOL 2811, BOR/MOL 2816, BOR/MOL 3049, BOR/MOL 6567, BOR/MOL 6570, BOR/MOL 6571, BOR/MOL 6574, BOR/MOL 6594, BOR/MOL 6612, BOR/MOL 6581, BOR/MOL 6583, BOR/MOL 6585, BOR/MOL 7860, BOR/MOL 7870,.BOR/MOL 7872, BOR/MOL 7879, BOR/MOL 8436, BOR/MOL 8442, BOR/MOL 8430, BOR/MOL 8515, BOR/MOL 8517, BOR/MOL 8596, BOR/MOL 8602, BOR/MOL 8604, BOR/MOL 11089, BOR/MOL 11102, BOR/MOL 11103, BOR/MOL 11113, BOR/MOL 11114, BOR/MOL 11115. *Pulau Gaya*: BOR/MOL 6302, BOR/MOL 6304, BOR/MOL 6616, BOR/MOL 6625, BOR/MOL 6626, BOR/MOL 6639, BOR/MOL 6640, BOR/MOL 6647, BOR/MOL 6649, BOR/MOL 6672, BOR/MOL 6676, BOR/MOL 6680, BOR/MOL 6682, BOR/MOL 8483, BOR/MOL 8485, BOR/MOL 8490, BOR/MOL 8506, BOR/MOL 8513, BOR/MOL 8522, BOR/MOL 8849, BOR/MOL 8853, BOR/MOL 8854, BOR/MOL 8855, BOR/MOL 8856, BOR/MOL 9721, BOR/MOL 10364, BOR/MOL 9729. *Pulau Rusukan Besar*: BOR/MOL 12268.

###### Distribution in Sabah.

*Island*: [West] Pulau Gaya, Pulau Tiga, Pulau Rusukan Besar; [North] Pulau Banggi, Pulau Balambangan; [East] Pulau Bod Gaya. *Mainland*: Kudat Division, West Coast Division, Interior Division, Sandakan Division, and Tawau Division.

###### Remarks.

Widespread in Sabah. Specimens from Pulau Rusukan Besar (BOR/MOL 12268) are probably fossilised.

##### Macrochlamys tersa

Taxon classificationAnimaliaStylommatophoraAriophantidae

(Issel, 1874)

Figure 11B

###### Type locality.

“Borneo” (Issel 1874)

###### Examined materials.

*Pulau Tiga*: BOR/MOL 2828, BOR/MOL 6577, BOR/MOL 6580, BOR/MOL 1062. *Pulau Mantanani Besar*: BOR/MOL 7171. *Pulau Labuan*: BOR/MOL 7928, BOR/MOL 12171, BOR/MOL 12193, BOR/MOL 12210. *Pulau Papan*: BOR/MOL 7825, BOR/MOL 7826, BOR/MOL 12060, BOR/MOL 12068, BOR/MOL 12069, BOR/MOL 12070. *Pulau Gaya*: BOR/MOL 9723. *Pulau Manukan*: BOR/MOL 10299. *Pulau Sapangar*: BOR/MOL 11998, BOR/MOL 11975, BOR/MOL 11985, BOR/MOL 11996.

###### Distribution in Sabah.

*Island*: [West] Pulau Tiga, Pulau Mantanani Besar, Pulau Labuan, Pulau Papan, Pulau Gaya, Pulau Manukan, Pulau Sapangar; [North] Pulau Banggi; [East] Pulau Bohey Dulang, Pulau Bod Gaya, Pulau Mantabuan, Pulau Sebangkat, Pulau Sibuan, Pulau Maiga. *Mainland*: Kudat Division, West Coast Division, Interior Division, Sandakan Division, and Tawau Division.

###### Remarks.

Widespread in Sabah.

##### Macrochlamys indica

Taxon classificationAnimaliaStylommatophoraAriophantidae

(Godwin-Austen, 1883)

Figure 11C

###### Type locality.

“India: West Bengal: Calcutta” (Godwin-Austen 1883)

###### Examined materials.

*Pulau Papan*: BOR/MOL 7821. *Pulau Labuan*: BOR/MOL 8591, BOR/MOL 12167, BOR/MOL 12169, BOR/MOL 12181, BOR/MOL 12186, BOR/MOL 12191, BOR/MOL 12209, BOR/MOL 12219, BOR/MOL 12220, BOR/MOL 12221, BOR/MOL 12226, BOR/MOL 12273, BOR/MOL 12488, BOR/MOL 12489, BOR/MOL 12490.

###### Distribution in Sabah.

*Island*: [West] Pulau Papan, Pulau Labuan. *Mainland*: West Coast Division, Interior Division.

###### Remarks.

Introduced to Sabah.

##### Microcystina striatula

Taxon classificationAnimaliaStylommatophoraAriophantidae

Vermeulen, Liew & Schilthuizen, 2015

Figure 11D

###### Type locality.

“Malaysia: Sabah: Lahad Datu–Tabin Valley” (Vermeulen et al. 2015)

###### Examined materials.

*Pulau Tiga*: BOR/MOL 1100. *Pulau Manukan*: BOR/MOL 10310. *Pulau Udar Kecil*: BOR/MOL 10370, BOR/MOL 10375. *Pulau Usukan*: BOR/MOL 12029, BOR/MOL 12033, BOR/MOL 12043, BOR/MOL 12054, BOR/MOL 12055, BOR/MOL 12470. *Pulau Papan*: BOR/MOL 12063, BOR/MOL 12065. *Pulau Rusukan Kecil*: BOR/MOL 12155. *Pulau Labuan*: BOR/MOL 12460.

###### Distribution in Sabah.

*Island*: [West] Pulau Tiga, Pulau Manukan, Pulau Udar Kecil, Pulau Usukan, Pulau Papan, Pulau Papan, Pulau Rusukan Kecil; [North] Pulau Banggi; [East] Pulau Bod Gaya, Pulau Sebangkat. *Mainland*: Kudat Division, West Coast Division, Interior Division, Sandakan Division, and Tawau Division.

###### Remarks.

Endemic to Borneo and widespread in Sabah.

##### Microcystina sinica

Taxon classificationAnimaliaStylommatophoraAriophantidae

Von Moellendorff, 1885

Figure 11E

###### Type locality.

“China: GuangDong Province–Shiu Heng Hap” (Von Moellendorff 1885)

###### Examined materials.

*Pulau Mantanani Besar*: BOR/MOL 3713, BOR/MOL 7168, BOR/MOL 7180, BOR/MOL 7184. *Pulau Mantanani Kecil*: BOR/MOL 3734. *Pulau Mengalum*: BOR/MOL 6175, BOR/MOL 12287, BOR/MOL 12313. *Pulau Tiga*: BOR/MOL 6602, BOR/MOL 8435, BOR/MOL 8424. *Pulau Gaya*: BOR/MOL 6620, BOR/MOL 6623, BOR/MOL 8452, BOR/MOL 8459, BOR/MOL 8473, BOR/MOL 8445, BOR/MOL 8505, BOR/MOL 8493, BOR/MOL 10365. *Pulau Dinawan*: BOR/MOL 8919, BOR/MOL 8901. *Pulau Usukan*: BOR/MOL 12031, BOR/MOL 12032, BOR/MOL 12041, BOR/MOL 12044, BOR/MOL 12047, BOR/MOL 12471, BOR/MOL 12478. *Pulau Labuan*: BOR/MOL 12185, BOR/MOL 12206.

###### Distribution in Sabah.

*Island*: [West] Pulau Mantanani Besar, Pulau Mantanani Kecil, Pulau Mengalum, Pulau Tiga, Pulau Gaya, Pulau Dinawan, Pulau Usukan, Pulau Labuan; [North] Pulau Banggi, Pulau Balambangan; [East] Pulau Bod Gaya, Pulau Bohey Dulang, Pulau Sebangkat, Pulau Selakan, Pulau Tetagan. *Mainland*: Kudat Division, West Coast Division, Interior Division, Sandakan Division, and Tawau Division.

###### Remarks.

Widespread in Sabah.

##### Microcystina microrhynchus

Taxon classificationAnimaliaStylommatophoraAriophantidae

Vermeulen, Liew & Schilthuizen, 2015

Figure 11F

###### Type locality.

“Malaysia: Sabah: Interior Province–Gua Pungiton” (Vermeulen et al. 2015)

###### Examined materials.

*Pulau Mantanani Besar*: BOR/MOL 3714. *Pulau Mantanani Kecil*: BOR/MOL 3733. *Pulau Gaya*: BOR/MOL 8453, BOR/MOL 8456, BOR/MOL 8463, BOR/MOL 8468, BOR/MOL 8474, BOR/MOL 8480, BOR/MOL 8508, BOR/MOL 8487, BOR/MOL 10367. *Pulau Mamutik*: BOR/MOL 10005. *Pulau Sapangar*: BOR/MOL 12001, BOR/MOL 11976, BOR/MOL 11982, BOR/MOL 11983, BOR/MOL 11986, BOR/MOL 11987, BOR/MOL 11988. *Pulau Labuan*: BOR/MOL 12207, BOR/MOL 12211.

###### Distribution in Sabah.

*Island*: [West] Mantanani group, Pulau Gaya, Pulau Mamutik, Pulau Sapangar, Pulau Labuan; [North] Pulau Banggi, Pulau Balambangan; [East] Pulau Bod Gaya, Pulau Bohey Dulang. *Mainland*: Kudat Division, West Coast Division, Interior Division, Sandakan Division, and Tawau Division.

###### Remarks.

Endemic to Borneo and widespread in Sabah.

##### Microcystina callifera

Taxon classificationAnimaliaStylommatophoraAriophantidae

Vermeulen, Liew & Schilthuizen, 2015

Figure 12A

###### Type locality.

“Malaysia: Sabah: Kota Belud–Mantanani Group, Pulau Lungisan” (Vermeulen et al. 2015)

**Figure 12.**
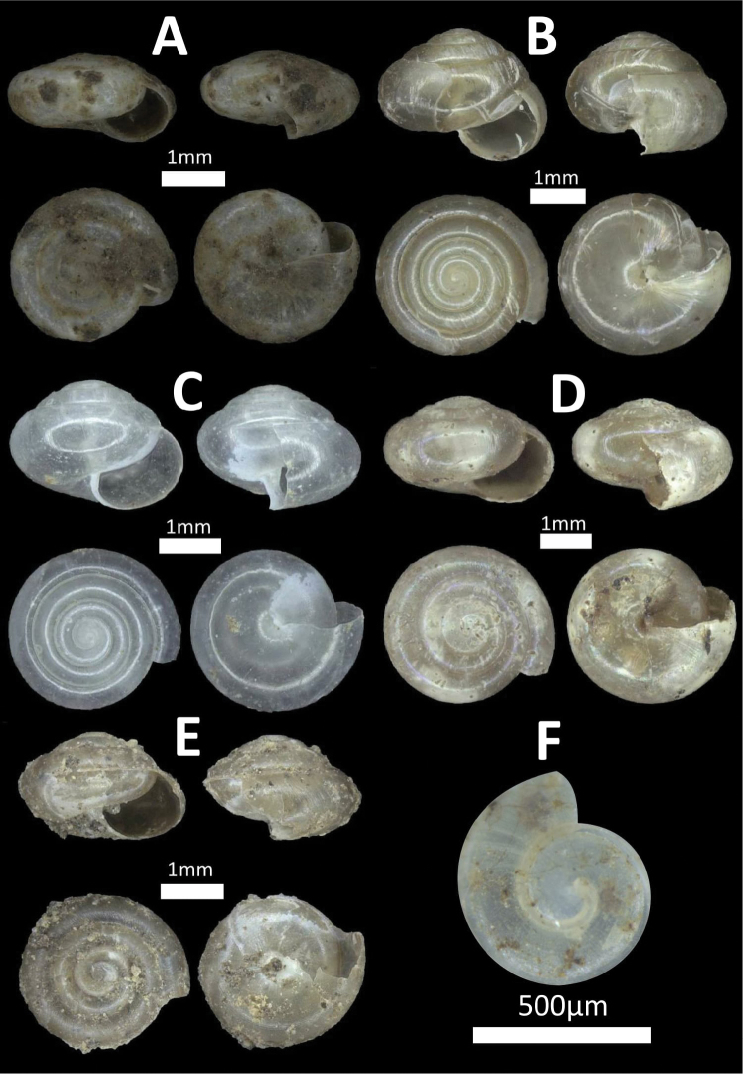
Family Ariophantidae. **A** *Microcystina callifera* (BOR/MOL 7176) **B** *Microcystina physotrochus* (BOR/MOL 8457) **C** *Microcystina physotrochus* (BOR/MOL 857) **D** *Microcystina muscorum* (BOR/MOL 8920) **E** *Microcystina* sp. 1 (BOR/MOL 1286) *Probably juvenile **F** *Microcystina* sp. 2 (BOR/MOL 10352) *Broken shell.

###### Examined materials.

*Pulau Lungisan*: BOR/MOL 3746, *Pulau Mantanani Besar*: BOR/MOL 3715, BOR/MOL 7159, BOR/MOL 7176, BOR/MOL 7186. *Pulau Mantanani Kecil*: BOR/MOL 3732.

###### Distribution in Sabah.

*Island*: [West] Mantanani group; [North] Pulau Banggi. *Mainland*: No record.

###### Remarks.

Endemic to Sabah. Only found in Pulau Mantanani Besar and Pulau Balambangan, which are close to the Palawan archipelago in the Philippines.

##### Microcystina physotrochus

Taxon classificationAnimaliaStylommatophoraAriophantidae

Vermeulen, Liew & Schilthuizen, 2015

Figure 12B, C

###### Type locality.

“Malaysia: Sabah: Sandakan–Kinabatangan Valley, Batu Keruak 2, near Sukau” (Vermeulen et al. 2015)

###### Examined materials.

*Pulau Gaya*: BOR/MOL 8451, BOR/MOL 8457, BOR/MOL 8464, BOR/MOL 8478, BOR/MOL 8482, BOR/MOL 8447, BOR/MOL 8507, BOR/MOL 8498. *Pulau Lungisan*: V 9862 (Not seen).

###### Distribution in Sabah.

*Island*: [West] Pulau Gaya, Pulau Lungisan. *Mainland*: West Coast Division, Interior Division, Sandakan Division, and Tawau Division.

###### Remarks.

Endemic to Borneo and widespread in Sabah. The specimen BOR/MOL 8457 consists of a single white, translucent shell (Figure 12C) which differs from other brown shells.

##### Microcystina muscorum

Taxon classificationAnimaliaStylommatophoraAriophantidae

van Benthem Jutting, 1959

Figure 12D

###### Type locality.

“Indonesia: North Sumatra: Karo Regency–Berastagi” (van Benthem Jutting 1959)

###### Examined materials.

*Pulau Dinawan*: BOR/MOL 8920, BOR/MOL 8903, BOR/MOL 8906, BOR/MOL 8907. *Pulau Manukan*: BOR/MOL 10306, BOR/MOL 10315, BOR/MOL 10323, BOR/MOL 10565. *Pulau Gaya*: BOR/MOL 10360. *Pulau Mantukod*: BOR/MOL 11066, BOR/MOL 10994, BOR/MOL 12465. *Pulau Tiga*: BOR/MOL 8439, BOR/MOL 8416, BOR/MOL 11080. *Pulau Sapangar*: BOR/MOL 11970, BOR/MOL 11995. *Pulau Labuan*: BOR/MOL 12179, BOR/MOL 12197. *Pulau Mengalum*: BOR/MOL 12286.

###### Distribution in Sabah.

*Island*: [West] Pulau Dinawan, Pulau Manukan, Pulau Mantukod, Pulau Gaya, Pulau Tiga, Pulau Sapangar, Pulau Labuan, Pulau Mengalum; [North] Pulau Banggi, Pulau Balambangan; [East] Pulau Bod Gaya, Pulau Bohey Dulang, Pulau Sibuan. *Mainland*: Kudat Division, West Coast Division, Interior Division, Sandakan Division, and Tawau Division.

###### Remarks.

Widespread in Sabah.

##### Microcystina

Taxon classificationAnimaliaStylommatophoraAriophantidae

sp. 1

Figure 12E

###### Type locality.

Not applicable.

###### Examined materials.

*Pulau Labuan*: BOR/MOL 12486.

###### Distribution in Sabah.

*Island*: [West] Pulau Labuan. *Mainland*: No record.

###### Remarks.

Morphology differs from others *Microcystina* species in Sabah which was revised recently by Vermeulen et al. (2015).

##### Microcystina

Taxon classificationAnimaliaStylommatophoraAriophantidae

sp. 2

Figure 12F

###### Type locality.

Not applicable

###### Examined materials.

*Pulau Peduk*: BOR/MOL 10352.

###### Distribution in Sabah.

*Island*: [West] Pulau Peduk. *Mainland*: No record.

###### Remarks.

Morphology differs from others *Microcystina* species in Sabah which was revised recently by Vermeulen et al. (2015).

#### FAMILY BRADYBAENIDAE

##### Bradybaena similaris

Taxon classificationAnimaliaStylommatophoraBradybaenidae

(Férussac, 1821)

Figure 13

###### Type locality.

“Indonesia: East Nusa Tenggara–Timor” (Férussac 1821)

**Figure 13.**
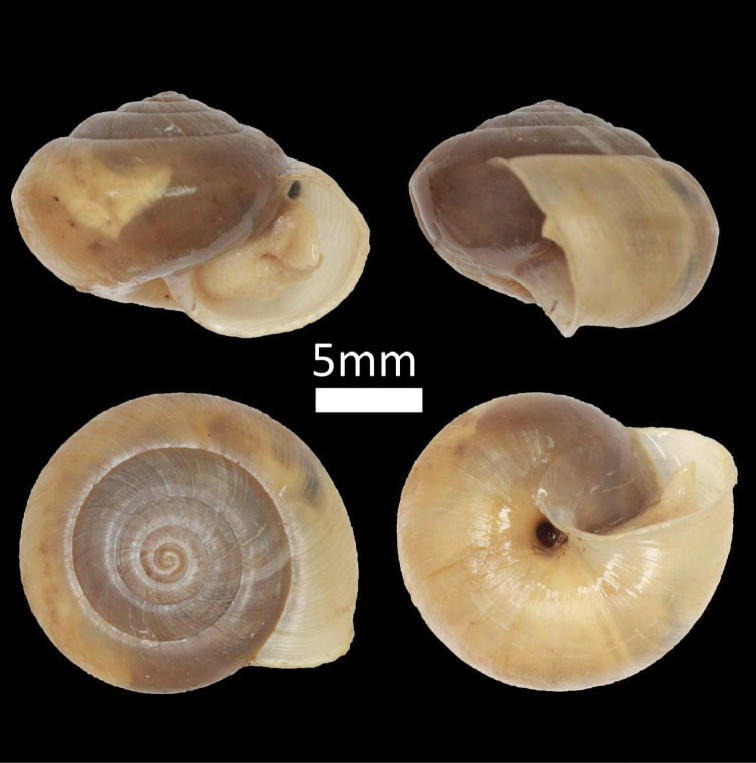
Family Bradybaenidae. *Bradybaena similaris* (BOR/MOL 6766).

###### Examined materials.

*Pulau Mamutik*: BOR/MOL 6299, BOR/MOL 6763, BOR/MOL 6766, BOR/MOL 10327, BOR/MOL 10564. *Pulau Labuan*: BOR/MOL 8669, BOR/MOL 8803, BOR/MOL 12165. *Pulau Kuraman*: BOR/MOL 8623, BOR/MOL 12125. *Pulau Sapangar*: BOR/MOL 12012.

###### Distribution in Sabah.

*Island*: [West] Pulau Mamutik, Pulau Labuan, Pulau Kuraman, Pulau Sapangar; [North] Pulau Banggi. *Mainland*: West Coast Division, Sandakan Division, and Tawau Division.

###### Remarks.

Introduced and widespread in Sabah.

#### FAMILY CAMAENIDAE

##### Amphidromus adamsi adamsi

Taxon classificationAnimaliaStylommatophoraCamaenidae

(Reeve, 1848)

Figure 14A

###### Type locality.

“Malaysia: Sabah: Kudat–Banguey island” (Fulton 1896)

**Figure 14.**
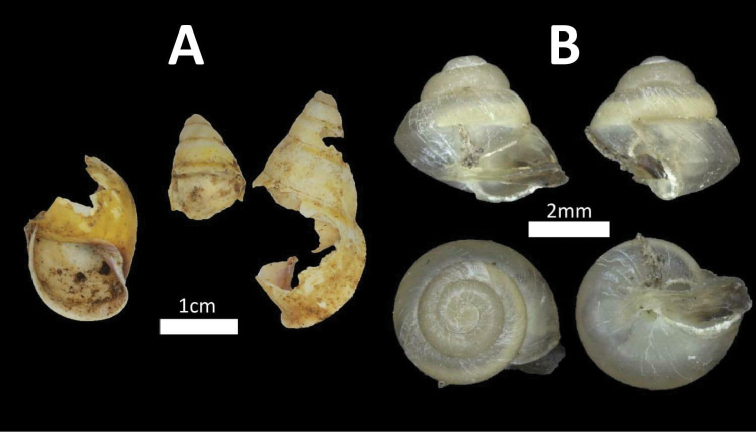
Family Camaenidae. **A** *Amphidromus adamsi adamsi* (BOR/MOL 3712) *Broken shell **B** *Ganesella tigaensis* (BOR/MOL 6573) *Juvenile.

###### Examined materials.

*Pulau Mantanani Besar*: BOR/MOL 3712, BOR/MOL 7192, BOR/MOL 7202. *Pulau Mantanani Kecil*: BOR/MOL 3735.

###### Distribution in Sabah.

*Island*: [West] Mantanani group; [North] Pulau Balambangan, Pulau Banggi. *Mainland*: No record.

###### Remarks.

Only found in Pulau Mantanani Besar and Pulau Balambangan, which are close to the Palawan archipelago in the Philippines. All specimens from the Mantanani Island group are broken shells.

##### Ganesella tigaensis

Taxon classificationAnimaliaStylommatophoraCamaenidae

(Godwin-Austen, 1891)

Figure 14B

###### Type locality.

“Malaysia: Sabah: Kuala Penyu–Tiga Island” (Godwin-Austen 1891)

###### Examined materials.

*Pulau Tiga*: BOR/MOL 6573.

###### Distribution in Sabah.

*Island*: [West] Pulau Tiga. *Mainland*: No record.

###### Remarks.

Endemic to Pulau Tiga.

#### FAMILY CHAROPIDAE

##### Discocharopa aperta

Taxon classificationAnimaliaStylommatophoraCharopidae

(Von Moellendorff, 1888)

Figure 15A

###### Type locality.

“Philippines: Rizal: Montalban” (Von Moellendorff 1888)

**Figure 15.**
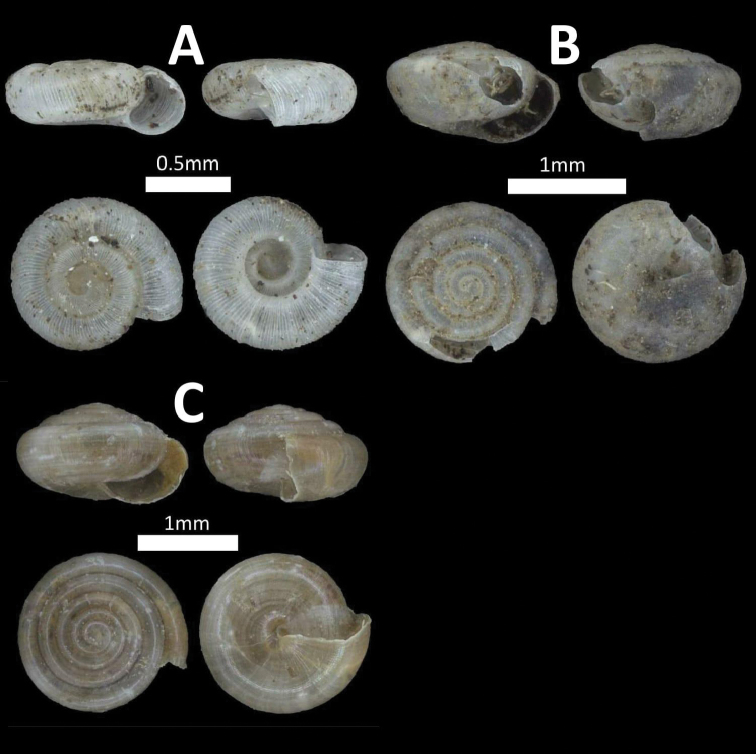
Family Charopidae. **A** *Discocharopa aperta* (BOR/MOL 11092) **B** *Charopa* sp “jugalis” (BOR/MOL 11989) **C** *Charopa* sp “lissobasis” (BOR/MOL 12180).

###### Examined materials.

*Pulau Mantanani Besar*: BOR/MOL 3711, BOR/MOL 7165, BOR/MOL 7175, BOR/MOL 7188. *Pulau Mantanani Kecil*: BOR/MOL 7199, BOR/MOL 3736. *Pulau Lungisan*: BOR/MOL 3747. *Pulau Tiga*: BOR/MOL 11092. *Pulau Kuraman*: BOR/MOL 12106. *Pulau Rusukan Besar*: BOR/MOL 12259.

###### Distribution in Sabah.

*Island*: [West] Mantanani Group, Pulau Tiga, Pulau Kuraman, Pulau Rusukan Besar; [North] Pulau Banggi, Pulau Balambangan; [East] Pulau Bod Gaya, Pulau Bohey Dulang, Pulau Selakan, Pulau Tetagan. *Mainland*: West Coast Division, and Tawau Division.

###### Remarks.

Widespread in Sabah.

##### Charopa

Taxon classificationAnimaliaStylommatophoraCharopidae

sp. “jugalis”

Figure 15B

###### Type locality.

Not applicable.

###### Examined materials.

*Pulau Sapangar*: BOR/MOL 11989.

###### Distribution in Sabah.

*Island*: [West] Pulau Sapangar. *Mainland*: Sandakan Division, Tawau Division and Interior Division.

###### Remarks.

Provisional working species name based on a manuscript in preparation. Endemic to Borneo. Widespread in Sabah. Only single shell found in Pulau Sapangar.

##### Charopa

Taxon classificationAnimaliaStylommatophoraCharopidae

sp. “lissobasis”

Figure 15C

###### Type locality.

Not applicable.

###### Examined materials.

Pulau Labuan: BOR/MOL 12180.

###### Distribution in Sabah.

*Island*: [West] Pulau Labuan. *Mainland*: Kudat Division, West Coast Division, Interior Division, Sandakan Division, and Tawau Division.

###### Remarks.

Provisional working species name based on a manuscript in preparation. Endemic and widespread in Sabah. Only single shell found in Pulau Labuan.

#### FAMILY DYAKIIDAE

##### Everettia jucunda

Taxon classificationAnimaliaPanpulmonataDyakiidae

(Pfeiffer, 1863)

Figure 16A

###### Type locality.

“Malaysia: Sabah: Labuan” (Pfeiffer 1863)

**Figure 16.**
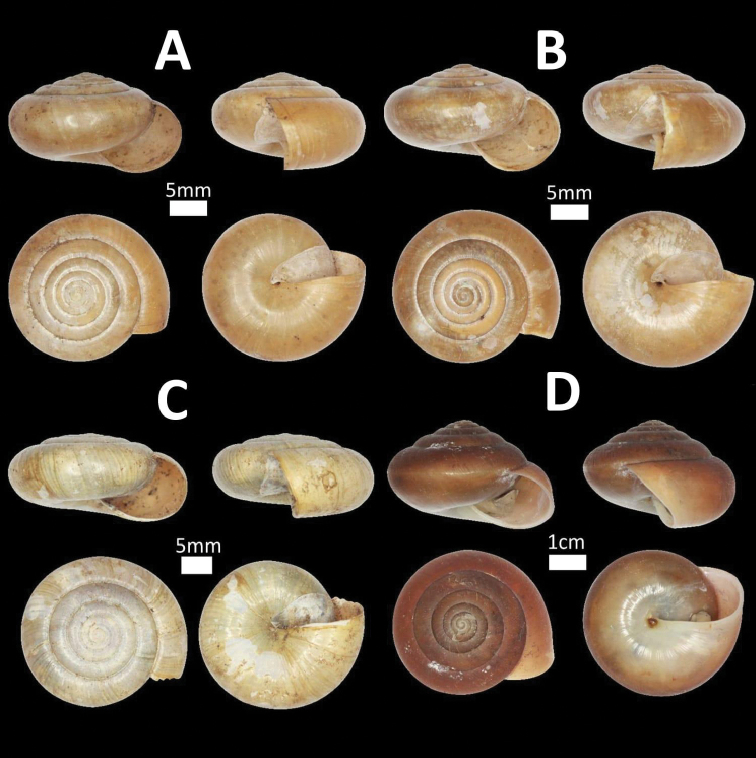
Family Dyakiidae. **A** *Everettia jucunda* (BOR/MOL 8419) **B** *Everettia subconsul* (BOR/MOL 12121) **C** *Everettia* sp. (BOR/MOL 12491) **D** *Quantula striata* (BOR/MOL 7923).

###### Examined materials.

*Pulau Tiga*: BOR/MOL 932, BOR/MOL 4237, BOR/MOL 4261, BOR/MOL 6566, BOR/MOL 6569, BOR/MOL 6601, BOR/MOL 6609, BOR/MOL 6611, BOR/MOL 6597, BOR/MOL 6576, BOR/MOL 6579, BOR/MOL 8413, BOR/MOL 8441, BOR/MOL 8443, BOR/MOL 8419, BOR/MOL 8514, BOR/MOL 8516, BOR/MOL 8520, BOR/MOL 8597, BOR/MOL 11088, BOR/MOL 11090, BOR/MOL 11095, BOR/MOL 11105, BOR/MOL 11111, BOR/MOL 11116. *Pulau Labuan*: BOR/MOL 6671, BOR/MOL 7912, BOR/MOL 7916, BOR/MOL 7918, BOR/MOL 7919, BOR/MOL 7920, BOR/MOL 8796, BOR/MOL 12225, BOR/MOL 12448, BOR/MOL 12450, BOR/MOL 12451, BOR/MOL 12453, BOR/MOL 12454, BOR/MOL 12457.

###### Distribution in Sabah.

*Island*: [West] Pulau Tiga, Pulau Labuan. *Mainland*: Interior Division.

###### Remarks.

Endemic to Borneo.

##### Everettia subconsul

Taxon classificationAnimaliaPanpulmonataDyakiidae

(Smith, 1887)

Figure 16B

###### Type locality.

“North Borneo” (Smith 1887)

###### Examined materials.

*Pulau Gaya*: BOR/MOL 6617, BOR/MOL 6642, BOR/MOL 6644, BOR/MOL 6645, BOR/MOL 6666, BOR/MOL 6667, BOR/MOL 6677, BOR/MOL 6679, BOR/MOL 8479, BOR/MOL 8491, BOR/MOL 8499, BOR/MOL 8510, BOR/MOL 8511, BOR/MOL 8521, BOR/MOL 8852, BOR/MOL 9719, BOR/MOL 10366, BOR/MOL 11117. *Pulau Mengalum*: BOR/MOL 6158, BOR/MOL 6167, BOR/MOL 12327, BOR/MOL 12329, BOR/MOL 12346, BOR/MOL 12348, BOR/MOL 12304, BOR/MOL 12347. *Pulau Sapangar*: BOR/MOL 6783, BOR/MOL 6785, BOR/MOL 6787, BOR/MOL 6790, BOR/MOL 6793, BOR/MOL 6795, BOR/MOL 6797, BOR/MOL 6779, BOR/MOL 6781, BOR/MOL 12007, BOR/MOL 12008, BOR/MOL 12009, BOR/MOL 11972, BOR/MOL 11973, BOR/MOL 11992, BOR/MOL 12010, BOR/MOL 12017, BOR/MOL 12018, BOR/MOL 12019, BOR/MOL 12020, BOR/MOL 12021. *Pulau Kuraman*: BOR/MOL 8613, BOR/MOL 8627, BOR/MOL 8628, BOR/MOL 8633, BOR/MOL 8637, BOR/MOL 8645, BOR/MOL 8648, BOR/MOL 8654, BOR/MOL 12104, BOR/MOL 12113, BOR/MOL 12119, BOR/MOL 12121, BOR/MOL 12122, BOR/MOL 12132, BOR/MOL 12133, BOR/MOL 12135, BOR/MOL 12141, BOR/MOL 12142, BOR/MOL 12146. *Pulau Rusukan Besar*: BOR/MOL 12264, BOR/MOL 12269.

###### Distribution in Sabah.

*Island*: [West] Pulau Gaya, Pulau Mengalum, Pulau Sapangar, Pulau Kuraman, Pulau Rusukan Besar; [North] Pulau Banggi, Pulau Balambangan. *Mainland*: West Coast Division, Interior Division, Sandakan Division, and Tawau Division.

###### Remarks.

Endemic and widespread in Sabah.

##### Everettia

Taxon classificationAnimaliaPanpulmonataDyakiidae

sp.

Figure 16C

###### Type locality.

Not applicable.

###### Examined materials.

*Pulau Labuan*: BOR/MOL 7905.

###### Distribution in Sabah.

*Island*: [West] Pulau Labuan. *Mainland*: No record.

###### Remarks.

Morphology found to differ from other known *Everettia* species in Sabah based on comparisons with materials from Liew et al. (2009).

##### Quantula striata

Taxon classificationAnimaliaPanpulmonataDyakiidae

(Gray, 1834)

Figure 16D

###### Type locality.

Not stated.

###### Examined materials.

*Pulau Labuan*: BOR/MOL 7923, BOR/MOL 7924, BOR/MOL 8812, BOR/MOL 8815, BOR/MOL 12163, BOR/MOL 12170, BOR/MOL 12182, BOR/MOL 12189, BOR/MOL 12190, BOR/MOL 12216, BOR/MOL 12447, BOR/MOL 12449, BOR/MOL 12452, BOR/MOL 12487. *Pulau Papan*: BOR/MOL 7819, BOR/MOL 7820, BOR/MOL 7829, BOR/MOL 7832, BOR/MOL 7833, BOR/MOL 12056, BOR/MOL 12057, BOR/MOL 12061, BOR/MOL 12067, BOR/MOL 12071, BOR/MOL 12074, BOR/MOL 12076, BOR/MOL 12077, BOR/MOL 12078.

###### Distribution in Sabah.

*Island*: [West] Pulau Labuan, Pulau Papan. *Mainland*: No record.

###### Remarks.

Introduced to Sabah. This is the first record of this species in Sabah.

#### FAMILY ELLOBIIDAE

##### Pythia chrysostoma

Taxon classificationAnimaliaArchaeopulmonataEllobiidae

Tapparone Canefri, 1883

Figure 17

###### Type locality.

“Indonesia: North Maluku: Ternate–near house of Sultan” (Tapparone Canefri 1883)

**Figure 17.**
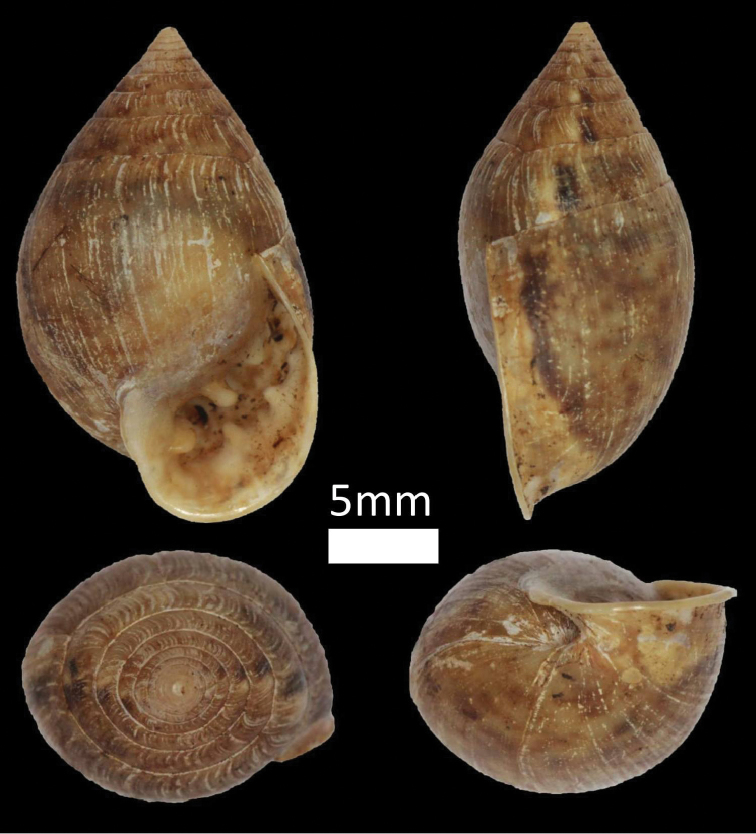
Family Ellobiidae. *Pythia chrysostoma* (BOR/MOL 12272).

###### Examined materials.

*Pulau Tiga*: BOR/MOL 6605, BOR/MOL 7866, BOR/MOL 8429, BOR/MOL 11106. *Pulau Gaya*: BOR/MOL 6619, BOR/MOL 6673, BOR/MOL 6690, BOR/MOL 8484, BOR/MOL 8512. *Pulau Sapangar*: BOR/MOL 6798, BOR/MOL 12003, BOR/MOL 12004, BOR/MOL 12016. *Pulau Udar Besar*: BOR/MOL 6801, BOR/MOL 11077, BOR/MOL 11078. *Pulau Mantanani Besar*: BOR/MOL 7167. *Pulau Mantanani Kecil*: BOR/MOL 7197. *Pulau Lungisan*: BOR/MOL 3751. *Pulau Dinawan*: BOR/MOL 7699. *Pulau Labuan*: BOR/MOL 8810, BOR/MOL 12164. *Pulau Mamutik*: BOR/MOL 10002, BOR/MOL 10010, BOR/MOL 10026. *Pulau Sulug*: BOR/MOL 10342, BOR/MOL 10347. *Pulau Manukan*: BOR/MOL 10343, BOR/MOL 10344. *Pulau Peduk*: BOR/MOL 10357. *Pulau Papan*: BOR/MOL 12062. *Pulau Mengalum*: BOR/MOL 12325, BOR/MOL 12277, BOR/MOL 12280, BOR/MOL 12281, BOR/MOL 12285. *Pulau Rusukan Kecil*: BOR/MOL 12158, BOR/MOL 12159. *Pulau Kuraman*: BOR/MOL 12137. *Pulau Rusukan Besar*: BOR/MOL 12272. *Pulau Usukan*: BOR/MOL 12275. *Pulau Burong*: BOR/MOL 12334.

###### Distribution in Sabah.

*Island*: [West] Mantanani group, Tunku Abdul Rahman Park, Labuan Marine Park, Pulau Tiga, Pulau Mengalum, Pulau Labuan, Pulau Papan, Pulau Usukan, Pulau Burong, Pulau Sapangar, Pulau Udar Besar; [North] Pulau Balambangan; [East] Pulau Bod Gaya, Pulau Sebangkat, Pulau Mantabuan, Pulau Sibuan, Pulau Maiga, Pulau Bohey Dulang. *Mainland*: Kudat Division, Tawau Division

###### Remarks.

Widespread in Sabah.

#### FAMILY EUCONULIDAE

##### Kaliella doliolum

Taxon classificationAnimaliaStylommatophoraEuconulidae

(Pfeiffer, 1846)

Figure 18A

###### Type locality.

“Philippines: Cebu: Sibonga” (Pfeiffer 1846)

**Figure 18.**
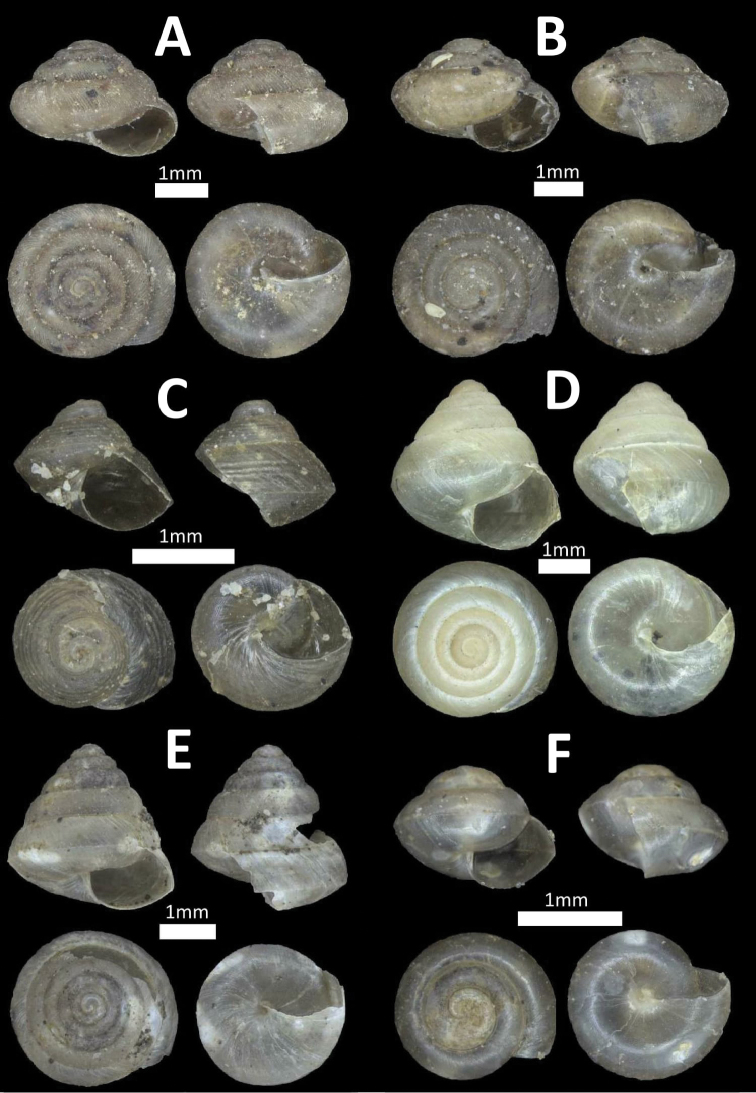
Family Euconulidae. **A** *Kaliella doliolum* (BOR/MOL 11994) **B** *Kaliella scandens* (BOR/MOL 6688) **C** *Kaliella dendrophila* (BOR/MOL 11971) *Juvenile **D** *Kaliella calculosa* (BOR/MOL 10989) **E** *Kaliella microconus* (BOR/MOL 12202) **F** *Kaliella barrakporensis* (BOR/MOL 12461) *Juvenile.

###### Examined materials.

*Pulau Tiga*: BOR/MOL 853, BOR/MOL 2701, BOR/MOL 8609, BOR/MOL 8610, BOR/MOL 6593, BOR/MOL 6600, BOR/MOL 6614, BOR/MOL 7862, BOR/MOL 7864, BOR/MOL 7865, BOR/MOL 8412, BOR/MOL 8433, BOR/MOL 8420, BOR/MOL 8599, BOR/MOL 8600, BOR/MOL 11082, BOR/MOL 11097. *Pulau Papan*: BOR/MOL 7823, BOR/MOL 7824. *Pulau Rusukan Besar*: BOR/MOL 8561, BOR/MOL 8565, BOR/MOL 8566, BOR/MOL 12230, BOR/MOL 12248, BOR/MOL 12261. *Pulau Kuraman*: BOR/MOL 8617, BOR/MOL 8618, BOR/MOL 8640, BOR/MOL 8652, BOR/MOL 8657, BOR/MOL 12100, BOR/MOL 12115, BOR/MOL 12129, BOR/MOL 12143. *Pulau Labuan*: BOR/MOL 8668, BOR/MOL 12455. *Pulau Dinawan*: BOR/MOL 8913, BOR/MOL 10991. *Pulau Sulug*: BOR/MOL 10329, BOR/MOL 10335. *Pulau Sapangar*: BOR/MOL 11999, BOR/MOL 11994. *Pulau Mengalum*: BOR/MOL 12298, BOR/MOL 12306, BOR/MOL 12318. *Pulau Pandan Pandan*: BOR/MOL 12468.

###### Distribution in Sabah.

*Island*: [West] Pulau Tiga, Pulau Papan, Pulau Rusukan Besar, Pulau Kuraman, Pulau Labuan, Pulau Dinawan, Pulau Sulug, Pulau Sapangar, Pulau Mengalum, Pulau Pandan Pandan; [North] Pulau Banggi, Pulau Balambangan; [East] Pulau Bohey Dulang, Pulau Tetagan, Pulau Mantabuan, Pulau Sibuan, Pulau Maiga. *Mainland*: Interior Division and Sandakan Division.

###### Remarks.

Widespread in Sabah.

##### Kaliella scandens

Taxon classificationAnimaliaStylommatophoraEuconulidae

(Cox, 1871)

Figure 18B

###### Type locality.

“Australia: New South Wales–Port Macquarie” (Cox 1871)

###### Examined materials.

*Pulau Mantanani Besar*: BOR/MOL 3710, BOR/MOL 7160, BOR/MOL 7190. *Pulau Mantanani Kecil*: BOR/MOL 3737, BOR/MOL 7198. *Pulau Mengalum*: BOR/MOL 6171, BOR/MOL 12309. *Pulau Gaya*: BOR/MOL 6621, BOR/MOL 6686, BOR/MOL 8465, BOR/MOL 8472, BOR/MOL 8448, BOR/MOL 8504, BOR/MOL 8509, BOR/MOL 8486, BOR/MOL 8497, BOR/MOL 9724. *Pulau Sapi*: BOR/MOL 6688, BOR/MOL 10290. *Pulau Sapangar*: BOR/MOL 6778, BOR/MOL 11980, BOR/MOL 12517. *Pulau Labuan*: BOR/MOL 7915, BOR/MOL 7921, BOR/MOL 7925, BOR/MOL 12198, BOR/MOL 12203, BOR/MOL 12351, BOR/MOL 12352. *Pulau Dinawan*: BOR/MOL 7686, BOR/MOL 7689, BOR/MOL 7690, BOR/MOL 9718. *Pulau Tiga*: BOR/MOL 8421. *Pulau Rusukan Kecil*: BOR/MOL 8553, BOR/MOL 8608, BOR/MOL 8547, BOR/MOL 8548, BOR/MOL 12152. *Pulau Rusukan Besar*: BOR/MOL 8563, BOR/MOL 8569, BOR/MOL 8570, BOR/MOL 8581, BOR/MOL 12232, BOR/MOL 12250. *Pulau Mamutik*: BOR/MOL 10004, BOR/MOL 10016, BOR/MOL 10020. *Pulau Manukan*: BOR/MOL 10313. *Pulau Sulug*: BOR/MOL 10337. *Pulau Peduk*: BOR/MOL 10349, BOR/MOL 10355. *Pulau Udar Kecil*: BOR/MOL 10372, BOR/MOL 10377. *Pulau Udar Besar*: BOR/MOL 11068, BOR/MOL 11073. *Pulau Mantukod*: BOR/MOL 10993, BOR/MOL 10997. *Pulau Usukan*: BOR/MOL 12034, BOR/MOL 12038, BOR/MOL 12046, BOR/MOL 12050, BOR/MOL 12476. *Pulau Papan*: BOR/MOL 12059.

###### Distribution in Sabah.

*Island*: [West] Pulau Mantanani Besar, Pulau Mantanani Kecil, Tunku Abdul Rahman Park, Pulau Mengalum, Pulau Sapangar, Pulau Labuan, Pulau Dinawan, Pulau Tiga, Pulau Rusukan Kecil, Pulau Rusukan Besar, Pulau Peduk, Pulau Udar Kecil, Pulau Udar Besar, Pulau Mantukod, Pulau Usukan, Pulau Papan; [North] Pulau Balambangan; [East] Pulau Bohey Dulang, Pulau Bod Gaya, Pulau Selakan. *Mainland*: Kudat Division, West Coast Division, Sandakan Division, Tawau Division and Interior Division.

###### Remarks.

Widespread in Sabah.

##### Kaliella dendrophila

Taxon classificationAnimaliaStylommatophoraEuconulidae

(van Benthem Jutting, 1950)

Figure 18C

###### Type locality.

“Indonesia: Java” (van Benthem Jutting 1950)

###### Examined materials.

*Pulau Sapangar*: BOR/MOL 6789, BOR/MOL 11971, BOR/MOL 11981, BOR/MOL 11990. *Pulau Mantukod*: BOR/MOL 7859, BOR/MOL 11070, BOR/MOL 12464. *Pulau Gaya*: BOR/MOL 8462, BOR/MOL 8476. *Pulau Dinawan*: BOR/MOL 9717, BOR/MOL 8904. *Pulau Sapi*: BOR/MOL 10296. *Pulau Manukan*: BOR/MOL 10321. *Pulau Mengalum*: BOR/MOL 12291.

###### Distribution in Sabah.

*Island*: [West] Pulau Sapangar, Pulau Mantukod, Pulau Gaya, Pulau Dinawan, Pulau Sapi, Pulau Manukan, Pulau Mengalum. *Mainland*: Interior Division and Sandakan Division.

###### Remarks.

Widespread in Sabah.

##### Kaliella calculosa

Taxon classificationAnimaliaStylommatophoraEuconulidae

(Gould, 1852)

Figure 18D

###### Type locality.

“France Polynesia: Society Islands–Tahiti” (Gould 1852)

###### Examined materials.

*Pulau Gaya*: BOR/MOL 6674, BOR/MOL 6675, BOR/MOL 10989. *Pulau Labuan*: BOR/MOL 7911, BOR/MOL 7902, BOR/MOL 7908, BOR/MOL 7909. *Pulau Kuraman*: BOR/MOL 8666.

###### Distribution in Sabah.

*Island*: [West] Pulau Gaya, Pulau Labuan, Pulau Kuraman. *Mainland*: Kudat Division, West Coast Division, Interior Division, Sandakan Division, and Tawau Division.

###### Remarks.

Widespread in Sabah.

##### Kaliella microconus

Taxon classificationAnimaliaStylommatophoraEuconulidae

(Mousson, 1865)

Figure 18E

###### Type locality.

“Fiji” (Mousson 1865)

###### Examined materials.

*Pulau Gaya*: BOR/MOL 9725, BOR/MOL 10361. *Pulau Labuan*: BOR/MOL 12202, BOR/MOL 12223.

###### Distribution in Sabah.

*Island*: [West] Pulau Gaya, Pulau Labuan; [North] Pulau Balambangan. *Mainland*: West Coast Division, Interior Division, Sandakan Division, and Tawau Division.

###### Remarks.

Widespread in Sabah.

##### Kaliella barrakporensis

Taxon classificationAnimaliaStylommatophoraEuconulidae

(Pfeiffer, 1852)

Figure 18F

###### Type locality.

“India: West Bengal: Kolkata–Barrakpore” (Pfeiffer 1852)

###### Examined materials.

*Pulau Labuan*: BOR/MOL 12199, BOR/MOL 12461.

###### Distribution in Sabah.

*Island*: [West] Pulau Labuan. *Mainland*: West Coast Division, Interior Division Sandakan Division, and Tawau Division.

###### Remarks.

Widespread in Sabah.

#### FAMILY STREPTAXIDAE

##### Huttonella bicolor

Taxon classificationAnimaliaStylommatophoraStreptaxidae

(Hutton, 1834)

Figure 19

###### Type locality.

“India: Uttar Pradesh: Mirzapur” (Hutton 1834)

**Figure 19.**
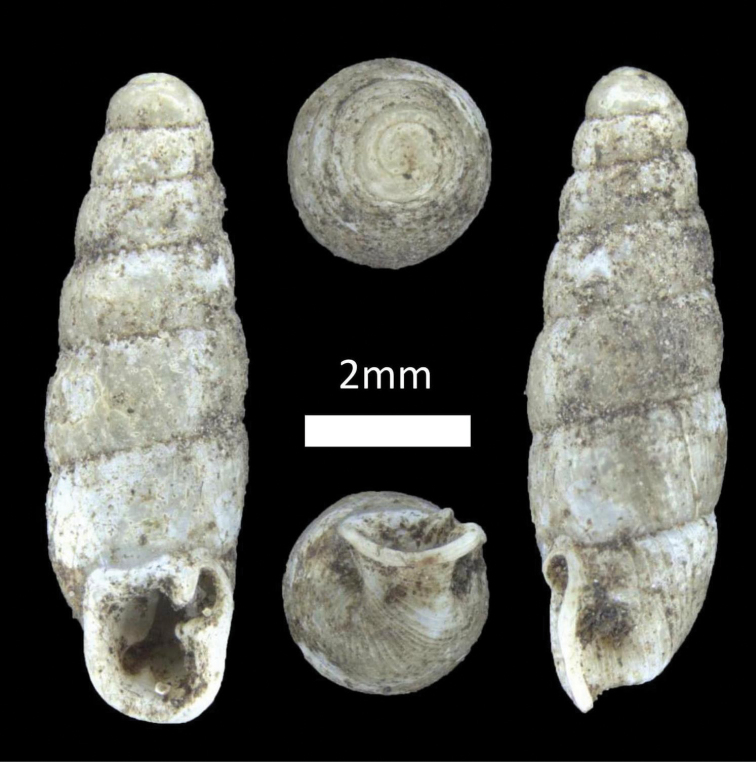
Family Streptaxidae. *Huttonella bicolor* (BOR/MOL 10374).

###### Examined materials.

*Pulau Labuan*: BOR/MOL 12174, BOR/MOL 12213, BOR/MOL 12458. *Pulau Mamutik*: BOR/MOL 10007, BOR/MOL 10022. *Pulau Udar Kecil*: BOR/MOL 10374. *Pulau Usukan*: BOR/MOL 12030, BOR/MOL 12473. *Pulau Rusukan Besar*: BOR/MOL 12233.

###### Distribution in Sabah.

*Island*: [West] Pulau Labuan, Pulau Mamutik, Pulau Udar Kecil, Pulau Usukan, Pulau Rusukan Besar. *Mainland*: Sandakan Division.

###### Remarks.

Introduced to Sabah.

#### FAMILY SUBULINIDAE

##### Paropeas achatinaceum

Taxon classificationAnimaliaStylommatophoraSubulinidae

(Pfeiffer, 1846)

Figure 20A

###### Type locality.

“Indonesia: Java” (Pfeiffer 1846)

**Figure 20.**
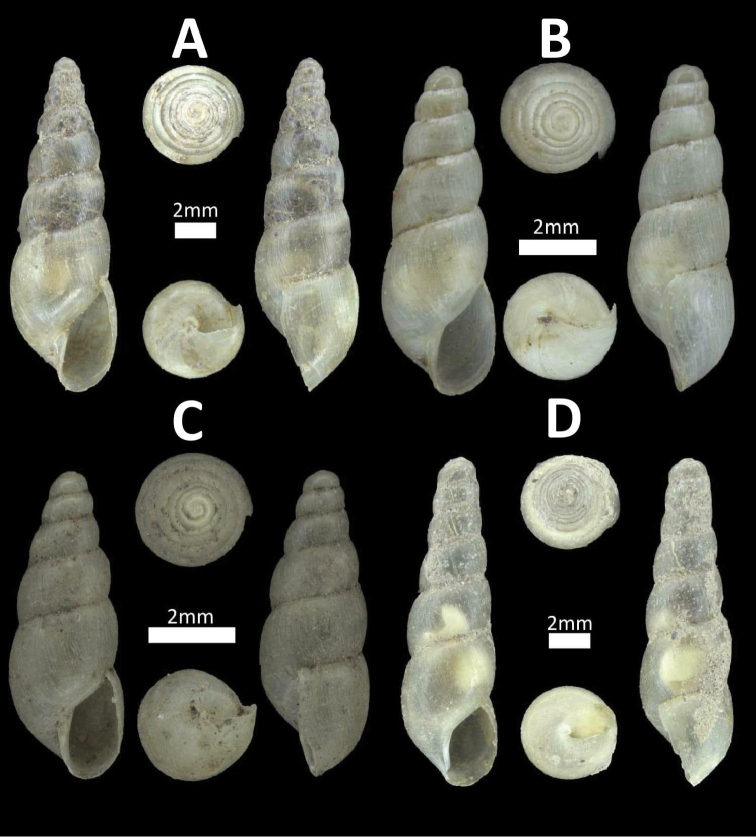
Family Subulinidae. **A** *Paropeas achatinaceum* (BOR/MOL 12518) **B** *Allopeas gracile* (BOR/MOL 12058) *Juvenile **C** *Allopeas clavulinum* (BOR/MOL 12539) **D** *Subulina octona* (BOR/MOL 12160).

###### Examined materials.

*Pulau Tiga*: BOR/MOL 11081, BOR/MOL 11099, BOR/MOL 11083. *Pulau Mantukod*: BOR/MOL 12463. *Pulau Sapangar*: BOR/MOL 11978. *Pulau Labuan*: BOR/MOL 12178, BOR/MOL 12187, BOR/MOL 12205, BOR/MOL 12208, BOR/MOL 12218, BOR/MOL 12222, BOR/MOL 8811. *Pulau Rusukan Besar*: BOR/MOL 12229, BOR/MOL 12238, BOR/MOL 12240, BOR/MOL 12244, BOR/MOL 12262. *Pulau Burong*: BOR/MOL 12337. *Pulau Rusukan Kecil*: BOR/MOL 12355. *Pulau Papan*: BOR/MOL 12518. *Pulau Dinawan*: BOR/MOL 8908, BORMOL, 8916, BOR/MOL 8921. *Pulau Gaya*: BOR/MOL 12540. *Pulau Kuraman*: BOR/MOL 12101, BOR/MOL 12118, BOR/MOL 12123, BOR/MOL 12140, BOR/MOL 12150. *Pulau Mantanani Besar*: BOR/MOL 7166. *Pulau Mengalum*: BOR/MOL 12292, BOR/MOL 12299, BOR/MOL 12308, BOR/MOL 12317, BOR/MOL 12319, BOR/MOL 12323. *Pulau Pandan Pandan*: BOR/MOL 10382, BOR/MOL 10383, BOR/MOL 12467, BOR/MOL 12469. *Pulau Peduk*: BOR/MOL 10354. *Pulau Sulug*: BOR/MOL 10334. *Pulau Udar kecil*: BOR/MOL 10371. *Pulau Usukan*: BOR/MOL 12035, BOR/MOL 12049, BOR/MOL 12474.

###### Distribution in Sabah.

*Island*: [West] Pulau Tiga, Pulau Mantukod, Pulau Sapangar, Pulau Labuan, Pulau Rusukan Besar, Pulau Burong, Pulau Rusukan Kecil, Pulau Papan, Pulau Dinawan, Pulau Gaya, Pulau Kuraman, Pulau Mantanani Besar, Pulau Mengalum, Pulau Pandan Pandan, Pulau Peduk, Pulau Sulug, Pulau Udar Kecil and Pulau Usukan. *Mainland*: West Coast Division, Interior Division and Tawau Division.

###### Remarks.

Widespread in Sabah.

##### Allopeas gracile

Taxon classificationAnimaliaStylommatophoraSubulinidae

(Hutton, 1834)

Figure 20B

###### Type locality.

“India: Uttar Pradesh: Mirzapur” (Hutton 1834)

###### Examined materials.

*Pulau Gaya*: BOR/MOL 10362, BOR/MOL 8454, BOR/MOL 8458, BOR/MOL 8467, BOR/MOL 8496, BOR/MOL 8503. *Pulau Kuraman*: BOR/MOL 12117, BOR/MOL 12130. *Pulau Labuan*: BOR/MOL 12188, BOR/MOL 12195, BOR/MOL 7914. *Pulau Mamutik*: BOR/MOL 10013, BOR/MOL 10021, BOR/MOL 9999. *Pulau Mantanani Besar*: BOR/MOL 3671, BOR/MOL 3709, BOR/MOL 7178, BOR/MOL 7189. *Pulau Mantanani Kecil*: BOR/MOL 3738, BOR/MOL 3758, BOR/MOL 7195. *Pulau Mantukod*: BOR/MOL 10995, BOR/MOL 10996. *Pulau Manukan*: BOR/MOL 10304, BOR/MOL 10312

BOR/MOL 10314, BOR/MOL 10317, BOR/MOL 10319, BOR/MOL 10322. *Pulau Mengalum*: BOR/MOL 12294, BOR/MOL 6160, BOR/MOL 6174. *Pulau Papan*: BOR/MOL 12058, BOR/MOL 12072, BOR/MOL 12073, BOR/MOL 12075, BOR/MOL 7822, BOR/MOL 7830, BOR/MOL 7831. *Pulau Rusukan Besar*: BOR/MOL 12231, BOR/MOL 12245, BOR/MOL 12258. *Pulau Rusukan Kecil*: BOR/MOL 12357. *Pulau Sapi*: BOR/MOL 10291, BOR/MOL 10297. *Pulau Sapangar*: BOR/MOL 11984, BOR/MOL 12002. *Pulau Sulug*: BOR/MOL 10328, BOR/MOL 10341. *Pulau Tiga*: BOR/MOL 6604, BOR/MOL 8415, BOR/MOL 8422, BOR/MOL 8437, BOR/MOL 8440, BOR/MOL 8598. Pulau Usukan: BOR/MOL 12042, BOR/MOL 12045, BOR/MOL 12479.

###### Distribution in Sabah.

*Island*: [West] Pulau Gaya, Pulau Kuraman, Pulau Labuan, Pulau Mamutik, Pulau Mantanani Besar, Pulau Mantanani Kecil, Pulau Mantukod, Pulau Manukan, Pulau Mengalum, Pulau Papan, Pulau Rusukan Besar, Pulau Rusukan Kecil, Pulau Sapi, Pulau Sapangar, Pulau Sulug, Pulau Tiga, Pulau Usukan; [North] Pulau Banggi, Pulau Balambangan; [East] Pulau Bohey Dulang, Pulau Bod Gaya, Pulau Sebangkat, Pulau Tetagan, Pulau Mantabuan, Pulau Sibuan, Pulau Maiga. *Mainland*: Kudat Division, West Coast Division, Interior Division, Sandakan Division, and Tawau Division.

###### Remarks.

Widespread in Sabah.

##### Allopeas clavulinum

Taxon classificationAnimaliaStylommatophoraSubulinidae

(Potiez & Michaud, 1838)

Figure 20C

###### Type locality.

“France: Reunion” (Potiez and Michaud 1838)

###### Examined materials.

*Pulau Tiga*: BOR/MOL 2292. *Pulau Burong*: BOR/MOL 12539. *Pulau Labuan*: BOR/MOL 12541.

###### Distribution in Sabah.

*Island*: [West] Pulau Tiga, Pulau Burong, Pulau Labuan. *Mainland*: Kudat Division, West Coast Division, Interior Division, Sandakan Division and Tawau Division.

###### Remarks.

Widespread in Sabah.

##### Subulina octona

Taxon classificationAnimaliaStylommatophoraSubulinidae

(Bruguière, 1789)

Figure 20D

###### Type locality.

“France: Antilles, Guadelupe, Dominican Republic” (Bruguière 1789)

###### Examined materials.

*Pulau Udar Kecil*: BOR/MOL 7157, BOR/MOL 7158, BOR/MOL 10378, BOR/MOL 10384. *Pulau Manukan*: BOR/MOL 6748, BOR/MOL 6757, BOR/MOL 6758, BOR/MOL 10300, BOR/MOL 10305, BOR/MOL 10308, BOR/MOL 10320, BOR/MOL 6759, BOR/MOL 10325. *Pulau Dinawan*: BOR/MOL 7682, BOR/MOL 8918, BOR/MOL 8900, BOR/MOL 8910. *Pulau Rusukan Kecil*: BOR/MOL 8606, BOR/MOL 12153, BOR/MOL 12156, BOR/MOL 12160, BOR/MOL 12151. *Pulau Kuraman*: BOR/MOL 8616, BOR/MOL 8624, BOR/MOL 8625, BOR/MOL 8629, BOR/MOL 8634, BOR/MOL 8643, BOR/MOL 12112, BOR/MOL 12116, BOR/MOL 12124, BOR/MOL 12128, BOR/MOL 12131, BOR/MOL 12138, BOR/MOL 12148, BOR/MOL 12149. *Pulau Labuan*: BOR/MOL 8667, BOR/MOL 12204, BOR/MOL 12215. *Pulau Mamutik*: BOR/MOL 10001, BOR/MOL 10012, BOR/MOL 10017, BOR/MOL 10019, BOR/MOL 10024, BOR/MOL 10326. *Pulau Sapi*: BOR/MOL 10289, BOR/MOL 10292, BOR/MOL 10294. *Pulau Mantukod*: BOR/MOL 10998, BOR/MOL 10999. *Pulau Papan*: BOR/MOL 12064.

###### Distribution in Sabah.

*Island*: [West] Pulau Udar Kecil, Pulau Manukan, Pulau Dinawan, Pulau Rusukan Kecil, Pulau Kuraman, Pulau Labuan, Pulau Mamutik, Pulau Sapi, Pulau Mantukod, Pulau Papan; [North] Pulau Banggi; [East] Pulau Sebangkat, Pulau Selakan, Pulau Mantabuan. *Mainland*: West Coast Division, Sandakan Division, Tawau Division.

###### Remarks.

Introduced and widespread in Sabah.

#### FAMILY TROCHOMORPHIDAE

##### Geotrochus conicoides

Taxon classificationAnimaliaStylommatophoraTrochomorphidae

(Metcalfe, 1851)

Figure 21A

###### Type locality.

“Borneo” (Metcalfe 1851)

**Figure 21.**
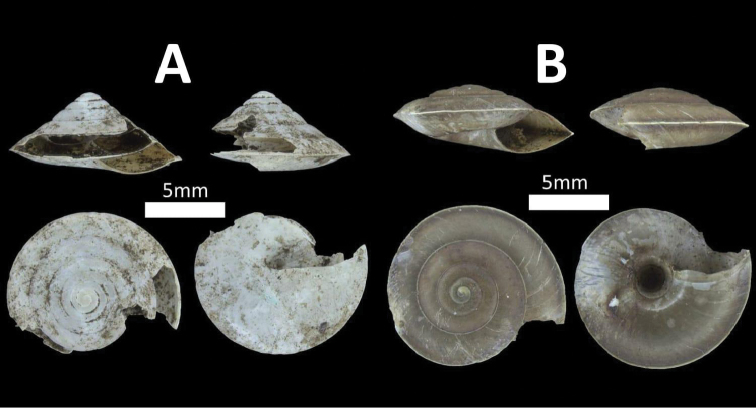
Family Trochomorphidae. **A** *Geotrochus conicoides* (BOR/MOL 899) *Broken shell **B** *Videna metcalfei* (BOR/MOL 12278).

###### Examined materials.

*Pulau Tiga*: BOR/MOL 899. *Pulau Gaya*: BOR/MOL 11119.

###### Distribution in Sabah.

*Island*: [West] Pulau Tiga, Pulau Gaya. *Mainland*: No record.

###### Remarks.

The sample from Pulau Tiga was collected in 2000 and not found in the current survey.

##### Videna metcalfei

Taxon classificationAnimaliaStylommatophoraTrochomorphidae

(Pfeiffer, 1845)

Figure 21B

###### Type locality.

“Philippine: Cebu” (Pfeiffer 1845)

###### Examined materials.

*Pulau Mengalum*: BOR/MOL 6161, BOR/MOL 6169, BOR/MOL 9992, BOR/MOL 9998, BOR/MOL 12283, BOR/MOL 12332, BOR/MOL 12350, BOR/MOL 12278, BOR/MOL 12293, BOR/MOL 12303, BOR/MOL 12311.

###### Distribution in Sabah.

*Island*: [West] Pulau Mengalum; [North] Pulau Banggi, Pulau Balambangan; [East] Pulau Bod Gaya, Pulau Bohey Dulang, Pulau Sebangkat, Pulau Selakan, Pulau Mantabuan, Pulau Selingaan. *Mainland*: Kudat Division, West Coast Division, Interior Division, Sandakan Division, and Tawau Division.

###### Remarks.

Widespread in Sabah.

#### FAMILY VERTIGINIDAE

##### Gastrocopta avanica

Taxon classificationAnimaliaStylommatophoraVertiginidae

(Benson, 1863)

Figure 22A

###### Type locality.

“Myanmar: Mandalay region: Kyaukse: Ava” (Benson 1863)

**Figure 22.**
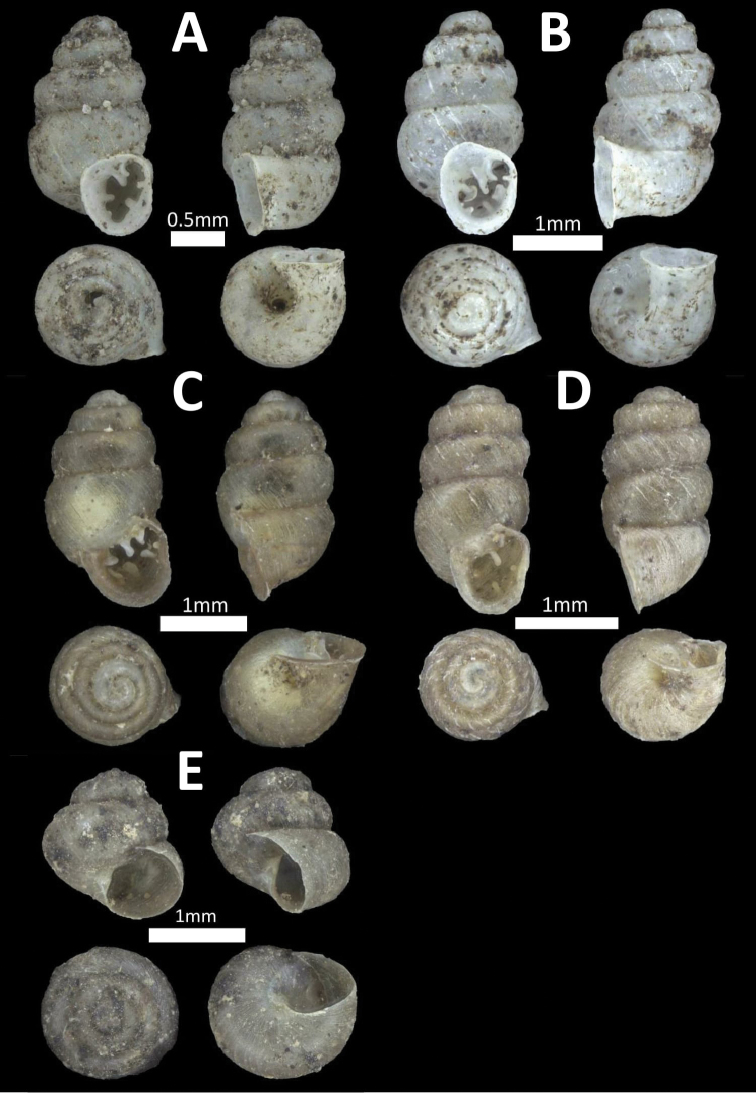
Family Vertiginidae. **A** *Gastrocopta avanica* (BOR/MOL 10376) **B** *Gastrocopta recondita* (BOR/MOL 8914) **C** *Nesopupa moreleti* (BOR/MOL 7182) **D** *Nesopupa malayana* (BOR/MOL 12324) **E** *Ptychopatula orcula* (BOR/MOL 11069).

###### Examined materials.

*Pulau Tiga*: BOR/MOL 565. *Pulau Mengalum*: BOR/MOL 6164, BOR/MOL 6173, BOR/MOL 8729, BOR/MOL 8861, BOR/MOL 8864, BOR/MOL 12321, BOR/MOL 12328, BOR/MOL 12349, BOR/MOL 12289, BOR/MOL 12310. *Pulau Udar Kecil*: BOR/MOL 10373, BOR/MOL 10376. *Pulau Rusukan Besar*: BOR/MOL 12257, BOR/MOL 12263. *Pulau Burong*: BOR/MOL 12335. *Pulau Pandan Pandan*: BOR/MOL 12466. *Pulau Labuan*: BOR/MOL 12217.

###### Distribution in Sabah.

*Island*: [West] Pulau Tiga, Pulau Mengalum, Pulau Udar Kecil, Pulau Rusukan Besar, Pulau Burong, Pulau Pandan Pandan, Pulau Labuan; [East] Pulau Selingaan. *Mainland*: Tawau Division.

###### Remarks.

Widespread in Sabah on islands.

##### Gastrocopta recondita

Taxon classificationAnimaliaStylommatophoraVertiginidae

(Tapparone Canefri, 1883)

Figure 22B

###### Type locality.

“Indonesia: Maluku–Aru Islands, Wokam” (Tapparone Canefri 1883)

###### Examined materials.

*Pulau Mantanani Kecil*: BOR/MOL 3739, BOR/MOL 7193, BOR/MOL 7194. *Pulau Mantanani Besar*: BOR/MOL 3708. *Pulau Lungisan*: BOR/MOL 3749. *Pulau Pandan Pandan*: BOR/MOL 7899. *Pulau Dinawan*: BOR/MOL 8914.

###### Distribution in Sabah.

*Island*: [West] Mantanani group, Pulau Dinawan, Pulau Pandan Pandan; [East] Pulau Mantabuan, Pulau Sibuan, Pulau Maiga. *Mainland*: No record.

###### Remarks.

Widespread in Sabah on islands.

##### Nesopupa moreleti

Taxon classificationAnimaliaStylommatophoraVertiginidae

(Brown, 1870)

Figure 22C

###### Type locality.

“Indonesia: Riau Islands–Natuna Island”

###### Examined materials.

*Pulau Tiga*: BOR/MOL 588, BOR/MOL 6603, BOR/MOL 11101. *Pulau Mantanani Besar*: BOR/MOL 7170, BOR/MOL 7182. *Pulau Dinawan*: BOR/MOL 7684, BOR/MOL 7691, BOR/MOL 12507. *Pulau Rusukan Besar*: BOR/MOL 8571, BOR/MOL 8580, BOR/MOL 12235, BOR/MOL 12251. *Pulau Kuraman*: BOR/MOL 8650, BOR/MOL 12110. *Pulau Mamutik*: BOR/MOL 10006, BOR/MOL 10015. *Pulau Sapi*: BOR/MOL 10295. *Pulau Sulug*: BOR/MOL 10340. *Pulau Peduk*: BOR/MOL 10351, BOR/MOL 10356. *Pulau Udar Kecil*: BOR/MOL 10381. *Pulau Udar Besar*: BOR/MOL 11072. *Pulau Usukan*: BOR/MOL 12036, BOR/MOL 12052, BOR/MOL 12472. *Pulau Sapangar*: BOR/MOL 11977.

###### Distribution in Sabah.

*Island*: [West] Pulau Tiga, Pulau Mantanani Besar, Pulau Dinawan, Pulau Rusukan Besar, Pulau Kuraman, Pulau Mamutik, Pulau Sapi, Pulau Sulug, Pulau Peduk, Pulau Udar Kecil, Pulau Udar Besar, Pulau Usukan, Pulau Sapangar; [North] Pulau Banggi; [East] Pulau Bohey Dulang. *Mainland*: No record.

###### Remarks.

Widespread in Sabah on islands.

##### Nesopupa malayana

Taxon classificationAnimaliaStylommatophoraVertiginidae

(Issel, 1874)

Figure 22D

###### Type locality.

“Borneo” (Issel 1874)

###### Examined materials.

*Pulau Mantanani Kecil*: BOR/MOL 3740. *Pulau Mengalum*: BOR/MOL 12324. *Pulau Rusukan Besar*: BOR/MOL 12256. *Pulau Labuan*: BOR/MOL 12459.

###### Distribution in Sabah.

*Island*: [West] Pulau Mantanani Kecil, Pulau Mengalum, Pulau Rusukan Besar, Pulau Labuan; [North] Pulau Banggi, Pulau Balambangan; [East] Pulau Bod Gaya, Pulau Bohey Dulang, Pulau Sebangkat. *Mainland*: No record.

###### Remarks.

Widespread in Sabah on islands.

##### Ptychopatula orcula

Taxon classificationAnimaliaStylommatophoraVertiginidae

(Benson, 1850)

Figure 22E

###### Type locality.

“India: Uttar Pradesh–between Jounpur and Benares” (Benson 1850)

###### Examined materials.

*Pulau Mantanani Besar*: BOR/MOL 3707, BOR/MOL 7162, BOR/MOL 7181. *Pulau Lungisan*: BOR/MOL 3750. *Pulau Mengalum*: BOR/MOL 6165, BOR/MOL 6172, BOR/MOL 8862, BOR/MOL 9994, BOR/MOL 12322, BOR/MOL 12290, BOR/MOL 12301, BOR/MOL 12307, BOR/MOL 12314. *Pulau Tiga*: BOR/MOL 6607, BOR/MOL 6613, BOR/MOL 8432, BOR/MOL 8426, BOR/MOL 8428, BOR/MOL 11098, BOR/MOL 10992. *Pulau Udar Besar*: BOR/MOL 6800, BOR/MOL 6805, BOR/MOL 6806, BOR/MOL 6807, BOR/MOL 11069, BOR/MOL 11071, BOR/MOL 11074. *Pulau Sulug*: BOR/MOL 6774, BOR/MOL 6775, BOR/MOL 6776, BOR/MOL 10331, BOR/MOL 10336. *Pulau Dinawan*: BOR/MOL 7687, BOR/MOL 7688, BOR/MOL 9716. *Pulau Papan*: BOR/MOL 7827, BOR/MOL 7828, BOR/MOL 7834, BOR/MOL 7836. *Pulau Gaya*: BOR/MOL 8469, BOR/MOL 8488. *Pulau Rusukan Besar*: BOR/MOL 8564, BOR/MOL 8572, BOR/MOL 8573, BOR/MOL 8587, BOR/MOL 12236, BOR/MOL 12239, BOR/MOL 12253. *Pulau Kuraman*: BOR/MOL 8619, BOR/MOL 8620, BOR/MOL 8641, BOR/MOL 8653, BOR/MOL 8665, BOR/MOL 12111, BOR/MOL 12147. *Pulau Mamutik*: BOR/MOL 10003, BOR/MOL 10011, BOR/MOL 10018. *Pulau Manukan*: BOR/MOL 10311, BOR/MOL 10990. *Pulau Peduk*: BOR/MOL 10350, BOR/MOL 10353. *Pulau Udar Kecil*: BOR/MOL 10380. *Pulau Rusukan Kecil*: BOR/MOL 12154, BOR/MOL 12353, BOR/MOL 12354. *Pulau Sapangar*: BOR/MOL 11979. *Pulau Labuan*: BOR/MOL 12196, BOR/MOL 12201.

###### Distribution in Sabah.

*Island*: [West] Pulau Mantanani Besar, Pulau Lungisan, Pulau Mengalum, Pulau Tiga, Pulau Udar Besar, Pulau Sulug, Pulau Dinawan, Pulau Gaya, Pulau Papan, Labuan Marine Park, Pulau Mamutik, Pulau Manukan, Pulau Peduk, Pulau Udar Kecil, Pulau Sapangar, Pulau Labuan; [North] Pulau Banggi, Pulau Balambangan; [East] Pulau Bohey Dulang, Pulau Mantabuan, Pulau Maiga, Pulau Bod Gaya. *Mainland*: Interior Division and Sandakan Division.

###### Remarks.

Widespread in Sabah.

## Author contributions

Conceived and designed the sampling method: TSL, CCP. Conducted the field samplings: CCP. Materials and logistics: LTS, PCC, FTYY. Compiled and analysed data: CCP, LTS. Wrote the paper: CCP, TSL.

## Supplementary Material

XML Treatment for Acmella striata

XML Treatment for Acmella polita

XML Treatment for Pterocyclos tenuilabiatus

XML Treatment for Pterocyclos amabilis

XML Treatment for Leptopoma pellucidum

XML Treatment for Platyraphe bongaoensis

XML Treatment for Japonia trilirata /kinabaluensis species complex

XML Treatment for Japonia balabacensis

XML Treatment for Japonia keppeli

XML Treatment for Ditropopsis koperbergi

XML Treatment for Ditropopsis imadatei

XML Treatment for Diplommatina recta

XML Treatment for Arinia borneensis

XML Treatment for Arinia

XML Treatment for Plectostoma jucundum

XML Treatment for Diplommatina

XML Treatment for Truncatella guerinii

XML Treatment for Truncatella marginata

XML Treatment for Aphanoconia usukanensis

XML Treatment for Georissa saulae

XML Treatment for Georissa scalinella

XML Treatment for Georissa williamsi

XML Treatment for Elasmias globulosum

XML Treatment for Elasmias manilense

XML Treatment for Tornatellinops moluccana

XML Treatment for Achatina fulica

XML Treatment for Hemiplecta humphreysiana

XML Treatment for Macrochlamys tersa

XML Treatment for Macrochlamys indica

XML Treatment for Microcystina striatula

XML Treatment for Microcystina sinica

XML Treatment for Microcystina microrhynchus

XML Treatment for Microcystina callifera

XML Treatment for Microcystina physotrochus

XML Treatment for Microcystina muscorum

XML Treatment for Microcystina

XML Treatment for Microcystina

XML Treatment for Bradybaena similaris

XML Treatment for Amphidromus adamsi adamsi

XML Treatment for Ganesella tigaensis

XML Treatment for Discocharopa aperta

XML Treatment for Charopa

XML Treatment for Charopa

XML Treatment for Everettia jucunda

XML Treatment for Everettia subconsul

XML Treatment for Everettia

XML Treatment for Quantula striata

XML Treatment for Pythia chrysostoma

XML Treatment for Kaliella doliolum

XML Treatment for Kaliella scandens

XML Treatment for Kaliella dendrophila

XML Treatment for Kaliella calculosa

XML Treatment for Kaliella microconus

XML Treatment for Kaliella barrakporensis

XML Treatment for Huttonella bicolor

XML Treatment for Paropeas achatinaceum

XML Treatment for Allopeas gracile

XML Treatment for Allopeas clavulinum

XML Treatment for Subulina octona

XML Treatment for Geotrochus conicoides

XML Treatment for Videna metcalfei

XML Treatment for Gastrocopta avanica

XML Treatment for Gastrocopta recondita

XML Treatment for Nesopupa moreleti

XML Treatment for Nesopupa malayana

XML Treatment for Ptychopatula orcula

## Supplementary materials

Supplementary material 1 Species list and occurrences Data type: occurrence Explanation note: An excel spreadsheet which provided following information: list of species, references for the species identification, occurrence of each species on every island and species richness of each island. This dataset is made available under the Open Database License (http://opendatacommons.org/licenses/odbl/1.0/). The Open Database License (ODbL) is a license agreement intended to allow users to freely share, modify, and use this Dataset while maintaining this same freedom for others, provided that the original source and author(s) are credited.Chee-Chean Phung, Fred Tuh Yit Yu, Thor-Seng Liew
